# Pulmonary Obstruction and Age, Not Activity, Associate With Muscle Oxidative Impairment in Smokers With and Without COPD

**DOI:** 10.1002/jcsm.70178

**Published:** 2026-01-19

**Authors:** Alessandra Adami, Fenghai Duan, Robert A. Calmelat, Zeyu Chen, Richard Casaburi, Harry B. Rossiter

**Affiliations:** ^1^ Department of Kinesiology, College of Health Sciences University of Rhode Island Kingston Rhode Island USA; ^2^ Department of Biostatistics and Center for Biostatistics and Health Data Science Brown University School of Public Health Providence Rhode Island USA; ^3^ Respiratory Research Center The Lundquist Institute for Biomedical Innovation at Harbor–UCLA Medical Center Torrance California USA; ^4^ Division of Respiratory and Critical Care, Physiology and Medicine Harbor–UCLA Medical Center Torrance California USA

**Keywords:** computed tomography, near‐infrared spectroscopy, physical activity, PRISm, smokers, triaxial accelerometry

## Abstract

**Background:**

Low muscle oxidative capacity is an extrapulmonary manifestation of chronic obstructive pulmonary disease (COPD) with unclear aetiology. We sought to characterize locomotor muscle oxidative capacity in never smokers and ever smokers with and without COPD and determine clinical and behavioural features associated with low muscle oxidative capacity.

**Methods:**

Two hundred forty‐three adults enrolled in the *Muscle Health Study*, an observational study ancillary to COPDGene. G*astrocnemius* oxidative capacity was measured by near‐infrared spectroscopy from the muscle oxygen consumption recovery rate constant (*k*). Physical activity was measured by accelerometry (vector magnitude units [VMU]/min). Pulmonary assessments included spirometry (FEV_1_%predicted), diffusing capacity (DL_CO_) and quantitative chest computed tomography (CT). Eighty‐seven variables related to COPD features were considered. Variables selected by univariate analysis of log‐transformed *k* with *p* ≤ 0.20 and filtered by machine learning were entered into multivariable linear regression to determine association with *k*.

**Results:**

Two hundred forty‐one (53.1% female; 45.6% African American; 64 ± 10 years old) participants were allocated to analysis. FEV_1_%predicted, DL_CO_, CT, pack‐years, age and VMU/min were among 24 variables selected by univariate analysis. After machine learning filtering on 162 (67%) cases with complete data, 11 variables were included in multivariable analysis. Only FEV_1_%predicted, age and race were significantly associated with *k* (*R* ^2^ = 0.26). Model coefficients equate a 10% lower FEV_1_%predicted to a 4.4% lower *k* or 10 years of aging to a 9.7% lower *k*. In 118 cases with CT available, FEV_1_%predicted and age remained associated with *k* (*R* ^2^ = 0.24). Physical activity was not retained in any model.

**Conclusions:**

Physical activity or radiographic COPD manifestations were not significantly associated with muscle oxidative impairment. Across never smokers and ever smokers with and without COPD, locomotor muscle oxidative capacity was positively associated with FEV_1_%predicted and negatively associated with age.

## 1 Introduction

In people with chronic obstructive pulmonary disease (COPD), impaired exercise capacity, skeletal muscle dysfunction and physical inactivity are strong predictors of increased healthcare utilization, poor quality of life and morbidity [1, 2, 3]. Extrapulmonary COPD manifestations, for example low bone density, muscle atrophy and weakness, low muscle oxidative capacity and capillarity, are also associated with mortality [2, 4]. Locomotor muscle structural changes (typically assessed in quadriceps) include atrophy, an increase in reactive oxygen species production and glycolytic Type II fibres, loss of fatigue‐resistant Type I fibres and ~10%–50% loss of oxidative capacity [2, 5, 6]. These adaptations may underlie the strong association between exercise intolerance and mortality in COPD [7, 8]. However, the aetiology of the muscular defect in oxidative function is still not well understood [3].

A large number of factors are proposed to contribute to loss of muscle oxidative capacity in COPD, from a normal deconditioning response to low physical activity (i.e., nonpathologic) to systemic factors (e.g., inflammation) related to smoking and/or pulmonary obstruction severity [7, 9]. Muscle disuse leads to many of the changes observed in COPD including muscle atrophy, reduced oxidative capacity and vasoreactivity [10]. It remains unclear whether behavioural or biologic processes in COPD contribute to the loss of the muscle oxidative phenotype [3].

Skeletal muscle oxidative capacity may be estimated noninvasively by near‐infrared spectroscopy (NIRS) [11]. This relies on a property of activated muscle mitochondria, that the recovery rate constant (*k*, min^−1^) of muscle oxygen consumption following contractions is directly proportional to muscle oxidative capacity. This concept has been validated in single isolated muscle fibres and in comparison with muscle biopsy and ^31^P magnetic resonance spectroscopy [12]. NIRS‐based methods directly assess muscle cellular capacity for oxidation under occlusion, and its measurement is therefore isolated from pathological circulatory or pulmonary derangements observed in COPD [11].

This study had two aims: (1) to characterize locomotor muscle oxidative capacity in never smokers and ever smokers with and without COPD and (2) to determine clinical and behavioural features associated with muscle oxidative capacity in these participants, hypothesizing that low physical activity and older age would associate with low muscle oxidative capacity. Then, we sought to perform two exploratory multivariate subanalyses. The first was to identify whether COPD phenotypes from chest computed tomography (CT) were associated with muscle oxidative capacity. The second was a stratified analysis based on COPD status to identify clinical and behavioural correlates of muscle oxidative capacity between participants with COPD and normal spirometry controls.

## 2 Methods

### 2.1 Participants

We enrolled 243 adults, of whom 218 were current or former smokers with ≥ 10 pack‐years smoking history. Recognizing that smokers without COPD do not represent a ‘healthy’ condition, 76 smokers with normal spirometry were used as a COPD comparator group in an attempt to control for the influence of smoking history. Twenty‐seven participants were never smokers (lifetime < 100 cigarettes, < 52 cigars or < 12‐oz. pipe tobacco smoked; see further details in the Supporting Information).

Smokers with normal spirometry and never smoker individuals had postbronchodilator forced expiratory volume in 1 s (FEV_1_) divided by forced vital capacity (FVC) ≥ 0.70 and FEV_1_ ≥ 80% predicted. Preserved ratio impaired spirometry (PRISm) individuals had FEV_1_/FVC ≥ 0.70 but FEV_1_ < 80% predicted. Participants with COPD had FEV_1_/FVC < 0.70. All participants were in stable state, with no exacerbation within 4 weeks of enrolment. Exclusion criteria included participation in pulmonary rehabilitation within 18 months, pregnancy or significant disease other than COPD that may (i) put the person at risk by participation; (ii) influence study results, such as ischemic heart disease, musculoskeletal or renal disease; or (iii) limit the individual's ability to comply with study protocol.

Participants were informed about study procedures and risks before giving written informed consent. The study was approved by the Institutional Review Board of The Lundquist Institute at Harbor–UCLA Medical Center (20403‐01).

### 2.2 Study Design

**FIGURE 1:**
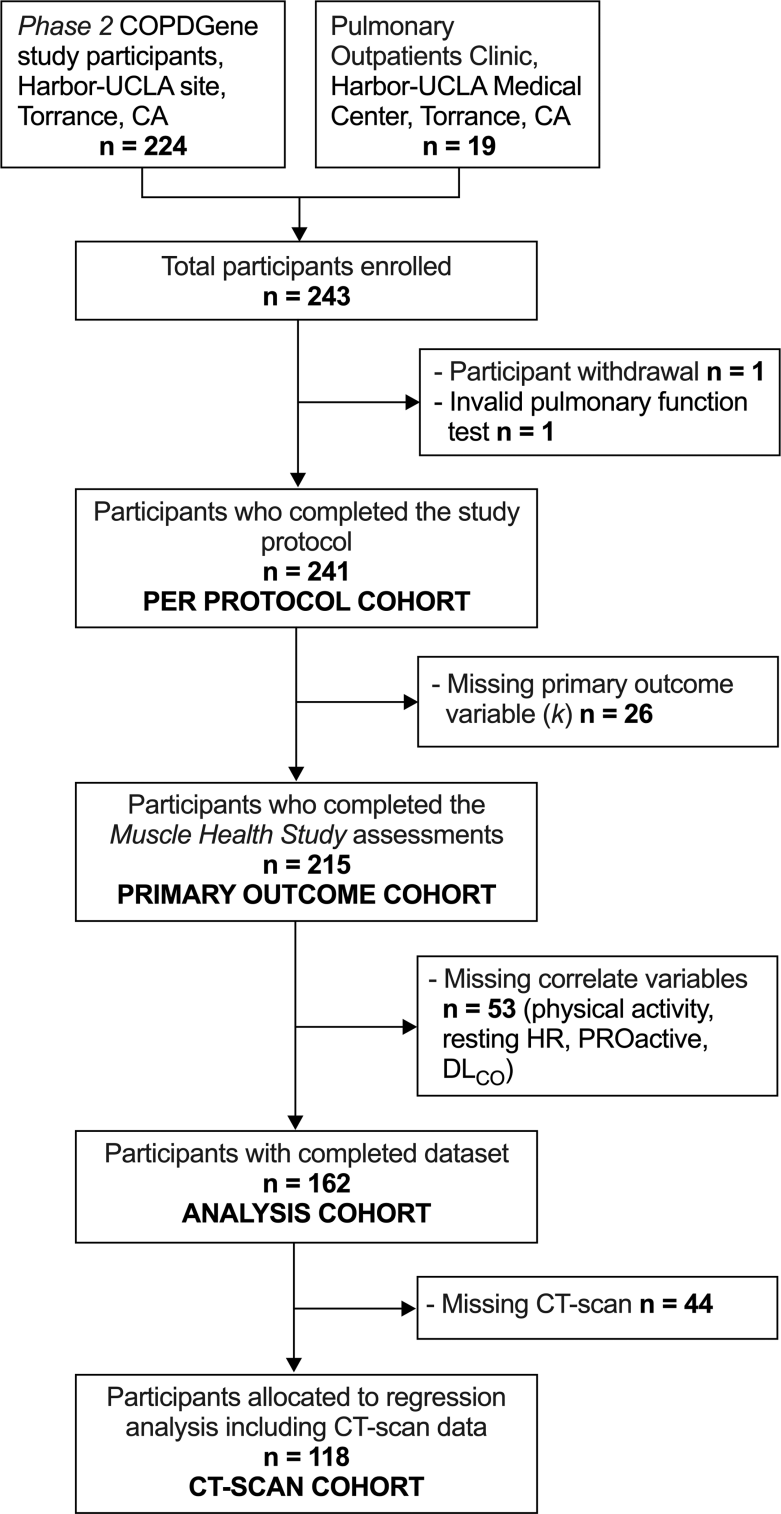
Study flow diagram. Study enrolment and allocation of participants to analysis.

The *Muscle Health Study*, a single‐centre observational study and ancillary study of COPDGene [13] (NCT00608764), took place between 2014 and 2016 at the Respiratory Research Center at the Lundquist Institute, Torrance, CA, USA. We enrolled 224 individuals who were also COPDGene participants, and 19 were recruited from Harbor–UCLA Medical Center pulmonary outpatient clinic (Figure 1).

Participants visited the laboratory once and completed, among other assessments: spirometry; lung diffusing capacity for carbon monoxide (DL_CO_); inspiratory and expiratory chest CT imaging; 6‐min walking distance (6MWD); questionnaires related to medical and smoking history, occupational and educational background, symptoms (COPD Assessment Test [CAT] and modified Medical Research Council Dyspnoea Scale [mMRC]), health‐related quality of life (St. George's Respiratory Questionnaire [SGRQ] and 36‐Item Short Form Health Survey [SF‐36]) and anxiety and depression (Hospital Anxiety and Depression Scale [HADS]); NIRS‐based muscle oxidative capacity; resting pulse oximetry monitoring; free‐living physical activity (PA) monitoring; and the Daily‐PROactive Physical Activity in COPD (D‐PPAC) instrument [14].

Detailed description of the methods is available in the Supporting Information.

### 2.3 Pulmonary Function and Imaging

Spirometry was performed in accordance with American Thoracic Society (ATS) guidelines [15]. DL_CO_, measured in accordance with European Respiratory Society/ATS standards [16], was adjusted for haemoglobin and altitude, and relative (%predicted) values were calculated using Global Lung Initiative reference equations [17].

Chest CT scans were performed in the supine position during a single full inspiration (200 mAs) and at end‐tidal exhalation (50 mAs) [13]. Airway wall thickening, %emphysema, %gas trapping and square root of wall area of a 10‐mm perimeter airway (Pi10) [18, 19, 20] were determined.

### 2.4 NIRS Muscle Oxidative Capacity

The medial *gastrocnemius* muscle was assessed using continuous‐wave, spatially resolved spectroscopy NIRS (PortaMon, Artinis BV, NL). NIRS measures relative concentration of deoxygenated (HHb + Mb) and oxygenated (HbO_2_ + MbO_2_) haemoglobin and myoglobin within the muscle up to a depth of ~1.5 cm under the probe. From these measurements, relative changes in total haemoglobin and myoglobin (THb = HHb + Mb + HbO_2_ + MbO_2_) are calculated. Tissue saturation index (TSI, an index of tissue oxygen saturation) was calculated using spatially resolved spectroscopy.

Participants lay supine on an exam bed for the NIRS test. Palpation during a series of brief isometric muscle contractions was used to identify optimal placement of the plastic‐wrapped NIRS probe [11] longitudinally over a highly activated region of the belly of the medial *gastrocnemius*. The NIRS probe was secured in position using an elastic bandage. A rapid‐inflation pressure cuff (SC12D, Hokanson, USA) was placed on the proximal thigh, on the same limb as the NIRS probe, and attached to an electronically controlled rapid cuff inflator (E20, Hokanson, USA). The participant was asked to relax and minimize limb movement, except when instructed. The participant was then familiarized with rapid cuff inflation and brief muscle contractions required in the protocol. Arterial occlusion pressure was determined from a tolerated cuff pressure within the range of 153–300 mmHg (mean: 221 ± 24 mmHg) that resulted in a rise in HHb + Mb, a fall in HbO_2_ + MbO_2_ and stable THb signal over 15–20 s. During the protocol, repeated dynamic muscle contractions were made at ~1 Hz by plantar flexion/relaxation against a light resistance (hereafter referred to as muscle contractions).

Initially, the participant laid at rest for 2–3 min to establish a stable baseline TSI. After this, ~10–15 s of light muscle contractions was performed to increase oxygen consumption of the muscle (mV̇O_2_), following which blood flow was occluded until a stable minimum TSI was reached or for 5 min (whichever occurred first). Cuff pressure was then released and the subsequent reactive reoxygenation monitored until resting baseline was re‐established (typically ~3 min [11]). This was used to determine an individualized muscle saturation physiological range (see Figure 1 in [11]). Finally, two muscle oxidative capacity assessments were performed, each consisting of (1) ~10‐ to 15‐s muscle contractions to increase mV̇O_2_ and desaturate the muscle to ~50% of the physiological range [11] and (2) a series of intermittent arterial occlusions (five occlusions for 5 s, 10 for 10 s, each separated by 5‐ to 20‐s recovery). Each of the assessments lasted ~6 min and were separated by ~2 min of rest.

For each intermittent arterial occlusion during oxidative capacity tests, the linear rate of decline in TSI (desaturation in %.s^−1^) was determined and a point value for relative mV̇O_2_ reported. Because the rate of tissue deoxygenation during arterial occlusion is inversely proportional to mV̇O_2_, its value is reported as positive (%.s^−1^; see Figure 2 in [11]). The primary study outcome was the exponential mV̇O_2_ recovery rate constant (*k*, min^−1^) estimated using non‐linear least‐squares regression [11] (OriginPro v8.6, OriginLab Co., Northampton, USA). This protocol has good test–retest reliability in smokers with and without COPD (ICC ≥ 0.88 [11, 12]).

### 2.5 Pulse Oximetry

Arterial oxygen saturation was estimated at rest prior to the NIRS test using fingertip pulse oximetry (SpO_2_) (Rad‐5 MasimoSET, Masimo Co., USA).

### 2.6 Free‐Living Daily Physical Activity

Participants underwent 7 days of PA monitoring using triaxial accelerometry (Dynaport MoveMonitor, McRoberts BV, NL). Each participant was instructed in the correct positioning of the monitor in the small of the back and to adjust the elastic waistband to ensure the device was in contact with the body and comfortable. Participants were asked to wear the PA monitor for as long as possible during each 24‐h period and to remove it only for bathing or swimming. The PA monitor was worn from the end of the study visit until seven full days had elapsed. Data were processed using the manufacturer's protocols.

Daily PA measurements were accepted as valid if they met the criteria that the monitor was worn for at least 8 h.day^−1^ during waking hours [14] and for at least 4 days.week^−1^ (not necessarily consecutive, without distinction between weekdays and weekends). Compliance with these conditions was 93%. PA is reported as the mean number of steps per day (total accumulated during each daytime period) and as the mean count of vector magnitude units per minute (VMU/min) during ‘daytime’ hours between 8 am and 11 pm [21]. VMU is the vectorial sum of movements in the three orthogonal planes (sagittal, frontal and transversal) measured over a 1‐min epoch. The device includes an accelerometer sensor sensitive to these orthogonal axes. The acceleration is measured while the device is worn, the signal is converted to a digital representation and processed to obtain an ‘activity count’, i.e., the VMU. Sample frequency of the triaxial accelerometer is automatically set to 100 Hz (for additional details on sensor technical characteristics and algorithms, see, e.g., [22]).

Subjective PA experience was determined using the D‐PPAC, on the same days the accelerometer was worn [14]. D‐PPAC results are presented as Rassh scores (0 [worst]–100 [best]) for subdomains of PA amount and difficulty and their sum total [14, 23].

## 3 Statistical Analyses

Data are presented as mean (SD) for continuous variables, counts and percentage for discrete variables. To evaluate group differences for continuous variables, we used Welch's ANOVA (robust to unequal variances and unequal group sizes). Games–Howell post hoc comparisons were performed using data from smokers with normal spirometry (here termed ‘Controls’) as the reference group. As part of post hoc analysis, we report effect sizes (mean differences vs. Controls) with 95% CIs for continuous variables whose overall tests were significant. Exact per‐group sample sizes are provided in the tables. For categorical variables, overall group differences were evaluated with Fisher's exact test using Monte Carlo–simulated *p* values (seven‐group comparison). For post hoc contrasts (each group vs. Controls), we used Fisher's exact test and controlled for multiplicity with a Bonferroni threshold of *α* = 0.05/6 across the six Controls‐based comparisons. In participants with normal spirometry, the effects of chronic smoking and of acute smoking (defined as smoking at least one cigarette within 2 h before the NIRS test) were evaluated. Group differences were assessed using Welch's ANOVA (Figure 3a) and Welch's *t* test (Figure 3b).

To determine variables associated with the recovery rate constant of muscle oxygen consumption (*k*; proportional to muscle oxidative capacity), a univariate linear regression analysis was performed on the Primary Outcome cohort (*n* = 215) with *k* log transformed to satisfy the homoscedasticity regression assumption. We evaluated model assumptions by inspecting diagnostic plots on both the original and log‐transformed scales (residuals vs. fitted, Q–Q, scale–location and residuals vs. leverage/Cook's distance) and selected the log transformation based on improved normality and variance constancy of residuals. Because the model is fit to log(*k*), effects are multiplicative on the original scale. Collinear variables, variables with more than 30% missing data and comorbidities with reported frequency of zero within the cohort (cancer of the kidney, cancer of the throat and mouth, connective tissue disorder, ovarian cancer and pancreatic cancer) were excluded from subsequent analysis. In total, 87 variables were considered in the univariate regression analysis, selected based on their likely ability to directly influence muscle oxidative capacity (a full list is provided in the Supporting Information).

Then, variables with *p* ≤ 0.20 in univariate linear regression were further filtered using machine learning–based feature selection methods (LASSO, random forest and XGBoost) to identify variables with the highest importance, i.e., the smallest cross‐validated mean‐squared error after machine learning filtering. These methods were selected to enhance the reliability of the identified variables and to account for more complex dependencies and interactions among correlates' effects on the response variable. A total of 13 correlates were identified to be included in the multivariable linear regression model. To address multicollinearity, two variables with high variance inflation factor (VIF) values were excluded. The remaining 11 top‐ranked variables were entered into the final multivariable linear regression to identify correlates of *k* in the Analysis cohort (*n* = 162).

Due to a relatively large number of participants who opted out of CT imaging (*n* = 44), a subset exploratory analysis was performed on those with a complete dataset that included CT imaging (CT Scan cohort; *n* = 118; Figure 1). A second exploratory analysis was on the same set of correlates stratified by COPD status, i.e., modelling participants with COPD or participants with normal spirometry separately. Additional details are provided in the Supporting Information. The significance level (*α*) was set at 0.05.

## 4 Results

### 4.1 Participants

As shown in Figure 1, of 243 enrolled, one withdrew, one was excluded for an invalid pulmonary function test and 241 (99.2%) completed study assessments (Per Protocol cohort). Table 1 reports the characteristics of this cohort and includes comparison across subgroups based on smoking history and severity of pulmonary obstruction.

**TABLE 1:** Participant characteristics included in the Per Protocol cohort (*n* = 241). Comparison is among smokers with normal spirometry (reference group; ‘Controls’), never smokers, preserved ratio impaired spirometry and participants with COPD (subdivided by disease severity stage).

	Per Protocol cohort	Never smokers	Normal spirometry smokers (Controls)	PRISm	COPD FEV_1_%predicted			
					> 80	50–79	30–49	< 30
*N*	241	26	76	21	30	40	35	13
**Personal characteristics**								
Age (years)	63.9 (9.8)	59.4 (10.3)	60.0 (8.7)	64.0 (11.2)	70.1 (9.6)^a^	65.7 (8.1)^a^	68.0 (8.0)^a^	65.2 (10.1)
Effect size		−0.62		3.99	10.10	5.73	8.02	5.22
[95% CI]		[−7.60, 6.36]		[−4.42, 12.40]	[3.88, 16.30]	[0.84, 10.60]	[2.94, 13.10]	[−4.80, 15.20]
Height (cm)	168.8 (9.8)	167.6 (8.3)	160.1 (9.7)	168.3 (9.3)	169.8 (10.5)	171.5 (9.6)	167.1 (11.2)	170.2 (9.5)
Weight (kg)	80.8 (18.6)	79.6 (15.3)	82.0 (20.8)	90.8 (18.6)	76.9 (15.3)	81.6 (16.9)	77.5 (19.4)	75.2 (17.8)
BMI (kg.m^−2^)	28.2 (5.9)	28.3 (4.4)	28.6 (6.7)	31.1 (5.7)	26.6 (4.1)	27.8 (5.3)	27.7 (6.3)	26.3 (7.2)
Sex (F/M)	128/113	15/11	42/34	14/7	17/13	18/22	18/17	4/9
Race (AA/As/Oth/W)	110/1/1/129	13/0/0/13	50/0/0/26	10/0/0/11	8/0/0/22^a^	13/0/0/27^a^	12/1/1/21^a^	4/0/0/9
Ethnicity (H/Not‐H)	3/238	0/26	0/76	0/21	0/30	0/40	2/33	1/12
**Pulmonary function, medical and smoking history**								
FVC (l)	3.1 (0.9)	3.5 (0.8)	3.3 (0.8)	2.3 (0.5)^a^	3.7 (0.8)	3.1 (0.6)	2.5 (0.8)^a^	2.2 (0.6)^a^
Effect size		0.17		−1.03	0.36	−0.19	−0.82	−1.12
[95% CI]		[−0.41, 0.76]		[−1.48, −0.59]	[−0.18, 0.91]	[−0.68, 0.29]	[−1.30, −0.33]	[−1.74, −0.49]
FEV_1_ (l)	2.1 (0.9)	2.9 (0.7)	2.7 (0.6)	1.8 (0.4)^a^	2.4 (0.6)	1.9 (0.5)^a^	1.0 (0.3)^a^	0.6 (0.2)^a^
Effect size		0.20		−0.90	−0.27	−0.79	−1.60	−2.04
[95% CI]		[−0.28, 0.68]		[−1.24, −0.56]	[−0.65, 0.11]	[−1.09, −0.48]	[−1.85, −1.35]	[−2.30, −1.79]
FEV_1_/FVC	0.66 (0.17)	0.81 (0.06)	0.80 (0.05)	0.76 (0.05)	0.64 (0.06)^a^	0.60 (0.06)^a^	0.43 (0.10)^a^	0.29 (0.09)^a^
Effect size		0.02		−0.04	−0.15	−0.20	−0.37	−0.51
[95% CI]		[−0.02, 0.06]		[−0.08, 0.004]	[−0.19, −0.12]	[−0.24, −0.16]	[−0.42, −0.31]	[−0.60, −0.42]
FEV_1_% pred	78.8 (28.5)	106.5 (13.8)	101.0 (12.5)	69.1 (10.9)^a^	92.9 (11.1)^a^	66.2 (10.0)^a^	40.0 (5.6)^a^	21.0 (4.3)^a^
Effect size		5.52		−31.80	−8.09	−34.80	−61.00	−80.00
[95% CI]		[−4.01, 15.10]		[−40.5, −23.1]	[−15.70, −0.54]	[−41.2, −28.3]	[−66.2, −55.9]	[−85.7, −74.3]
DL_CO_% pred	70.4 (22.6)	88.8 (10.8)	80.1 (17.0)	65.8 (13.2)^a^	75.5 (19.5)	66.2 (24.1)^a^	44.3 (12.6)^a^	37.2 (14.4)^a^
Effect size		8.78		−14.20	−4.55	−13.80	−35.70	−42.80
[95% CI]		[−0.20, 17.80]		[−25.30, −3.09]	[−17.30, 8.21]	[−27.40, −0.27]	[−45.6, −25.9]	[−58.2, −27.4]
Haemoglobin (g.dL^−1^)	13.7 (1.4)	13.6 (1.2)	13.7 (1.3)	13.5 (1.4)	13.7 (1.1)	13.9 (1.5)	13.7 (1.7)	14.2 (1.7)
Resting SpO_2_ (%)	97.5 (2.3)	98.7 (0.5)	98.4 (1.1)	97.1 (3.0)	97.6 (2.2)	97.4 (1.3)^a^	96.3 (2.3)^a^	94.5 (5.5)
Effect size		0.34		−1.26	−0.72	−0.96	−2.09	−3.89
[95% CI]		[−0.14, 0.82]		[−3.43, 0.91]	[−2.06, 0.62]	[−1.69, −0.22]	[−3.37, −0.81]	[−9.27, 1.48]
Supplementary O_2_, *N* (%)	26 (10.8)	0 (0)	0 (0)	1 (5)	1 (3)	1 (3)	14 (40)^a^	12 (92)^a^
Smoking duration (years)	37.5 (10.1)	—	34.4 (9.3)	39.0 (13.1)	38.9 (10.6)	39.8 (9.0)	41.5 (9.7)^a^	33.9 (8.2)
Effect size		—		4.56	4.52	5.38	7.08	−0.55
[95% CI]		—		[−5.16, 14.30]	[−2.36, 11.40]	[−0.06, 10.80]	[0.67, 13.50]	[−8.87, 7.77]
Smoking history (pack‐years)	44.4 (22.7)	—	39.4 (19.8)	43.6 (25.7)	39.8 (18.4)	52.7 (25.2)	52.9 (20.9)	41.9 (28.6)
Effect size		—		4.23	0.42	13.30	13.50	2.55
[95% CI]		—		[−15.10, 23.50]	[−12.10, 13.00]	[−0.79, 27.40]	[−0.09, 27.20]	[−25.60, 30.70]
Smoking status (NS/CS/FS)	27/82/132	26/0/0^a^	0/42/34	0/10/11	0/9/20	0/13/27	0/8/27^a^	0/0/13^a^
Severe exacerbation, *N* (%)	31 (13.1)	3 (11.5)	4 (5.3)	0 (0.0)	0 (0.0)	7 (17.5)	12 (37.5)^a^	5 (41.7)^a^
Frequency severe exacerbation (*N* median [min–max])	0 [0–6]	0 [0–1]	0 [0–3]	0 [0–2]	0 [0–2]	0 [0–2]	0 [0–3]^a^	0 [0–6]
Effect size		−0.03		0.08	0.00	0.26	0.47	1.02
[95% CI]		[−0.21, 0.16]		[−0.29, 0.44]	[−0.25, 0.25]	[−0.09, 0.61]	[0.0003, 0.93]	[−0.87, 2.90]
**Functional performance and activity**								
BODE	1.7 (2.2)	0.4 (0.8)	0.9 (1.2)	1.6 (1.8)	0.5 (1.1)	1.7 (1.5)	5.2 (1.5)^a^	6.9 (2.0)^a^
Effect size		−0.46		0.69	−0.36	0.79	4.31	6.01
[95% CI]		[−1.09, 0.17]		[−0.62, 2.00]	[−1.09, 0.36]	[−0.06, 1.63]	[3.32, 5.30]	[3.51, 8.51]
6MWD (m)	396 (107)	496 (95)^a^	400 (81)	380 (97)	445 (107)	401 (70)	324 (109)^a^	249 (105)^a^
Effect size		96.10		−20.83	44.30	0.39	−76.26	−151.14
[95% CI]		[31.4, 160.8]		[−93.8, 52.1]	[−22.5, 111.1]	[−43.2, 43.9]	[−139.5, −13.0]	[−260.2, −42.1]
Recent exercise, *N* (%)	135 (56.3)	20 (76.9%)	41 (54.0%)	12 (57.1%)	18 (60.0%)	25 (62.5%)	13 (38.2%)	6 (46.2%)
D‐PPAC total score	62.3 (12.5)	72.0 (13.1)	65.3 (10.1)	62.3 (8.0)	65.7 (11.6)	64.9 (10.5)	52.5 (9.0)^a^	47.1 (14.1)^a^
Effect size		6.86		−3.05	0.42	−0.33	−12.70	−18.10
[95% CI]		[−3.11, 16.80]		[−10.8, 4.74]	[−7.72, 8.56]	[−7.17, 6.51]	[−19.20, −6.33]	[−32.00, −4.26]
D‐PPAC amount score	48.5 (13.6)	53.7 (16.0)	51.7 (12.0)	49.7 (9.0)	52.8 (10.8)	49.3 (12.3)	40.9 (11.2)^a^	31.5 (15.5)^a^
Effect size		2.02		−1.96	1.11	−2.36	−10.80	−20.20
[95% CI]		[−10.10, 14.10]		[−10.80, 6.93]	[−6.97, 9.19]	[−10.40, 5.68]	[−18.60, −2.90]	[−35.60, −4.70]
D‐PPAC difficulty score	76.1 (17.5)	89.9 (13.9)^a^	78.2 (16.3)	74.1 (18.0)	77.9 (16.8)	79.9 (17.5)	63.5 (12.0)^a^	62.1 (15.1)^a^
Effect size		11.70		−4.14	−0.28	1.71	−14.70	−16.10
[95% CI]		[0.22, 23.20]		[−20.80, 12.50]	[−12.40, 11.80]	[−9.59, 13.00]	[−24.00, −5.51]	[−31.60, −0.65]

*Note:* Data are presented as mean (SD) for continuous variables and as counts (frequencies) for categorical variables if not otherwise specified. Spirometric variables are postbronchodilator. Overall tests: Welch's ANOVA (continuous) and Fisher's exact (categorical; Monte Carlo *p* values). Effect sizes and 95% CIs are shown only when the corresponding overall test is significant.

Abbreviations: 6MWD, 6‐min walking distance (in metres); AA, African American; As, Asian; BMI, body mass index; BODE, index derived from BMI, airflow obstruction, dyspnoea and exercise (6‐min walk distance); CS, current smoker; DL_CO_, lung diffusing capacity for carbon monoxide adjusted for [haemoglobin] and altitude; D‐PPAC, Daily‐PROactive Physical Activity instrument in COPD (score 0–100); F, female; FEV_1_, forced expiratory volume in 1 s; FS, former smoker; FVC, forced vital capacity; H, Hispanic or Latino; M, male; Not‐H, Not Hispanic or Latino; NS, never smoker; Oth, other races; Recent exercise, defined as having done exercise in the past 3 weeks (yes/no); Severe exacerbation, defined as having ≥ 2 severe exacerbations in the past 12 months (yes/no); SpO_2_, oxygen saturation; W, White Caucasian.

^a^ For continuous variables, Games–Howell post hoc *p* < 0.05 versus Controls and a significant overall test. For categorical variables, Fisher's exact post hoc *p* < 0.05/6 (Bonferroni) versus Controls and a significant overall test.

Among the 241 participants, 128 (53.1%) were female, 110 (45.6%) were of African American race and 3 (1.2%) identified as Hispanic ethnicity. Participants were 64 ± 10 years old and overweight (BMI 28.2 ± 5.9 kg.m^−2^). Thirty‐four per cent were current smokers, 54.8% were former smokers and 10.8% were never smokers. One hundred fifty (62.2%) participants had one or more comorbidities (Table S1), where hypertension (50.2%), high cholesterol (36.1%) and osteoarthritis (15.4%) were the most prevalent. Twenty‐six (10.8%) were prescribed supplementary O_2_ therapy. Also, 134 (55.6%) reported participation in exercise, defined as walking or biking for exercise at least twice a week within the previous 3 weeks.

Overall, there were 26 never smokers, 76 Controls, 21 PRISm and 118 COPD (Table 1). COPD participants were older than Controls, had significantly lower resting SpO_2_ and exercise performance (6MWD) and worse dyspnoea symptoms and quality of life (BODE, mMRC, CAT and SGRQ total score) (*p* < 0.01; Table S2). Never smokers had significantly greater 6MWD and reported less difficulty with PA (D‐PPAC difficulty score) compared with Controls.

Eight participants with COPD required nasal cannula O_2_ during the study visit (2–4 L.min^−1^; note that this has been found to not influence NIRS‐based muscle oxidative capacity assessment [11]).

### 4.2 Muscle Oxidative Capacity

Muscle oxidative capacity was reliably assessed in 215 (89.2%) participants. Twenty were unable to tolerate the pressure for arterial occlusions, while *k* could not be determined from recorded measurements in six tests.

**FIGURE 2:**
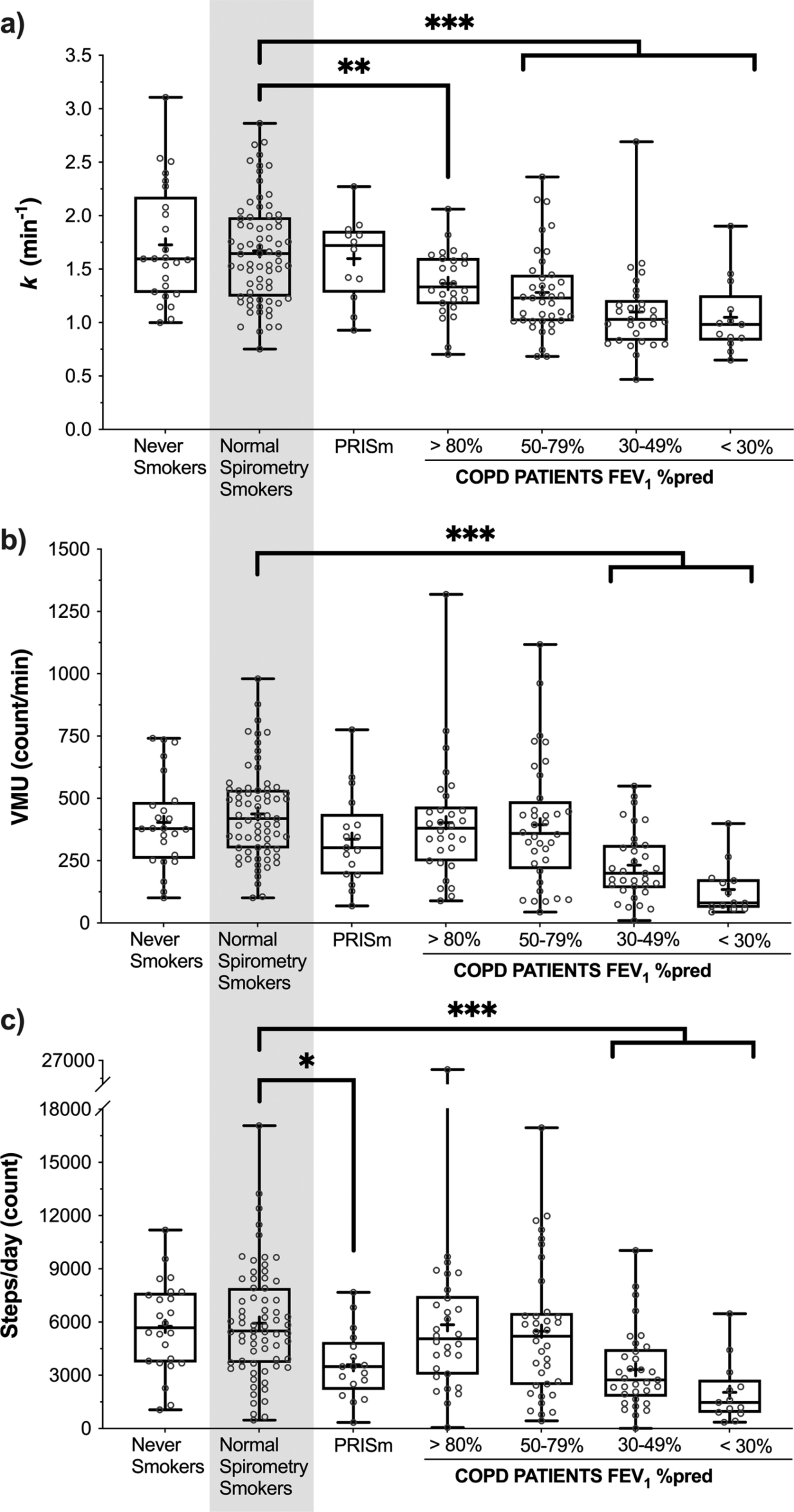
Comparison of (a) the rate constant of muscle oxygen consumption recovery (*k*; proportional to muscle oxidative capacity; *n* = 215), (b) physical activity (mean vector magnitude units per minute; *n* = 222) and (c) number of steps per day (*n* = 222), among smokers with normal spirometry (reference group; Controls), never smokers and participants with PRISm and COPD. COPD, chronic obstructive pulmonary disease; FEV_1_%pred, forced expiratory volume in 1 s as a percentage of the predicted value; *k*, rate constant of muscle oxygen consumption recovery; PRISm, preserved ratio impaired spirometry; VMU, vector magnitude units. *p < 0.05; **p < 0.01; ***p < 0.001.

Figure 2a shows that *k* was not different among never smokers (1.73 ± 0.55 min^−1^; *n* = 26), Controls (1.67 ± 0.48 min^−1^; *n* = 70) and PRISm (1.60 ± 0.39 min^−1^; *n* = 12). Compared with Controls, *k* was 17.9% lower in mild COPD (1.37 ± 0.31 min^−1^; *n* = 26; *p* < 0.003) and was progressively lower with increasing COPD severity (moderate 1.28 ± 0.40 min^−1^, *n* = 39; severe 1.10 ± 0.40 min^−1^, *n* = 29; very severe 1.05 ± 0.35 min^−1^, *n* = 13; each *p* < 0.0001).

**FIGURE 3:**
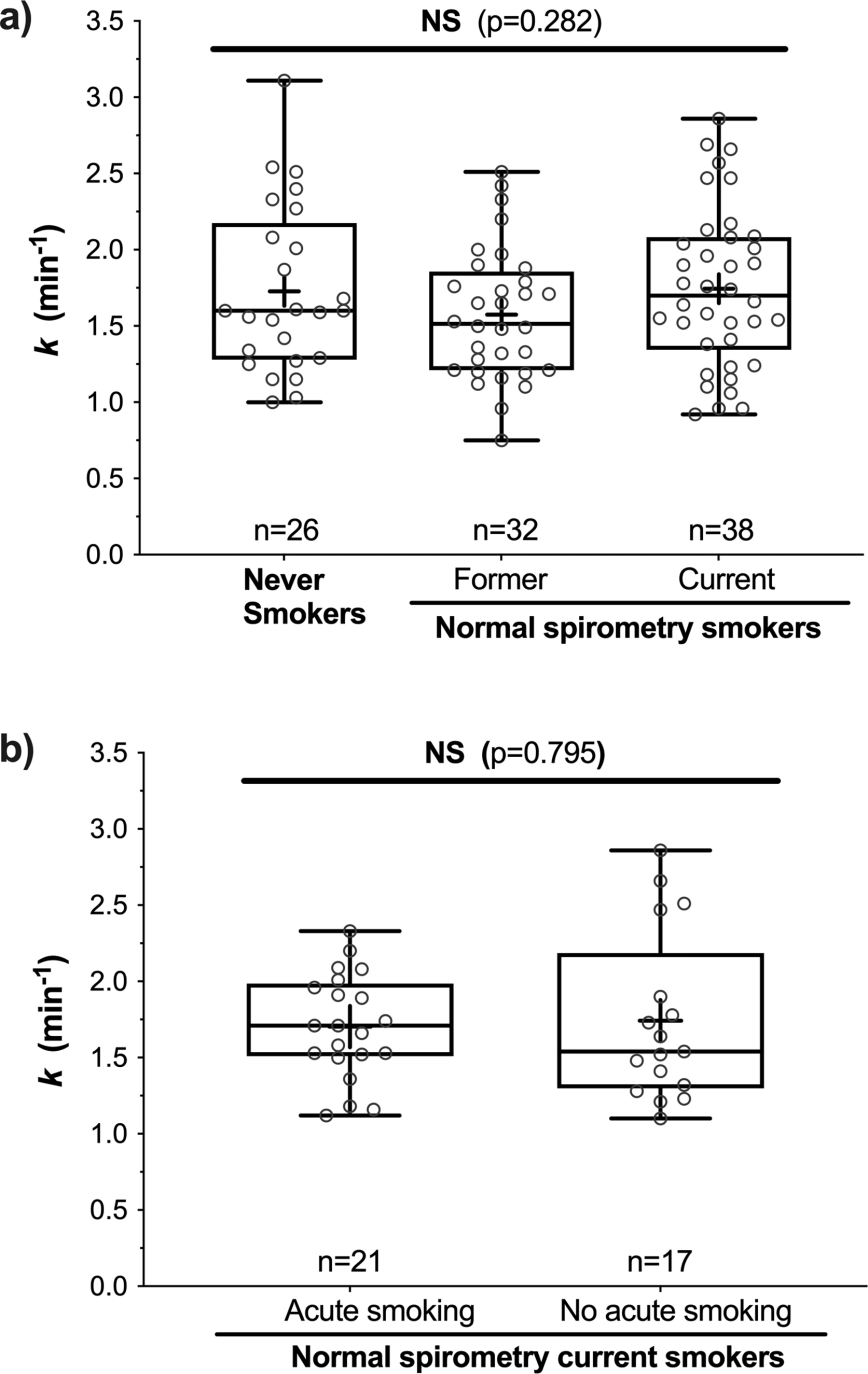
Chronic (a) and acute (b) effects of smoking on the rate constant of muscle oxygen consumption recovery (*k*; proportional to muscle oxidative capacity) in participants with normal spirometry. Data are from the Never Smoker (*n* = 26) and Normal Spirometry Smoker (Controls) groups (*n* = 70), where four current and two former smokers were missing data on acute smoking.

To address whether lower *k* in COPD was related to direct effects of smoke exposure rather than COPD per se, we investigated the acute effect of smoking on *k* by comparing (i) the effect of smoking within the 2 h prior to the NIRS test and (ii) the chronic effect of smoking (> 10 pack‐years) in smokers with normal spirometry in comparison with never smokers. We found no significant difference in *k* (*p* = 0.282) among never smokers (1.73 ± 0.55 min^−1^; *n* = 26), current smokers (1.75 ± 0.52 min^−1^, *n* = 38) or former smokers (1.58 ± 0.43 min^−1^, *n* = 32) with normal spirometry (Figure 3a). Within normal spirometry current smokers (*n* = 70), we found no significant difference in *k* (*p* = 0.795) between individuals who had smoked within 2 h (1.70 ± 0.34 min^−1^, *n* = 21) and those who had not (1.74 ± 0.55 min^−1^, *n* = 17) (Figure 3b).

### 4.3 Free‐Living Daily Physical Activity and Symptoms

Two hundred twenty‐three (92.5%) participants wore the triaxial accelerometer for at least eight daytime hours over an average of 5.6 ± 1.4 days. As expected, PA was lowest in severe COPD, who walked ≥ 44% fewer steps/day (severe 3328 ± 2241, *n* = 33; very severe 2037 ± 1756, *n* = 13; *p* < 0.0001) and moved ≥ 47% less on average (VMU/min: severe 232 ± 139 counts/min, *n* = 33; very severe 135 ± 73 counts/min, *n* = 13; *p* < 0.0001) than Controls (5938 ± 3121 steps/day; 438 ± 184 counts/min, *n* = 69) (Figure 2b,c). Of interest, steps/day were also lower in PRISm compared with Controls (3599 ± 1928, *n* = 17; *p* = 0.0022; Figure 2c). Otherwise, as shown in Figure 2b,c, PA behaviours in never smokers, mild and moderate COPD did not differ from Controls.

Participants with severe COPD reported lower amount, difficulty and total score using D‐PPAC compared with Controls (*p* < 0.01; Table 1), indicating greater difficulty in sustaining the limited activity they performed. Similarly, physical functioning and physical component scores of SF‐36 were significantly (*p* < 0.001) lower in those with severe COPD compared with Controls (Table S2).

Severe COPD participants reported worse symptoms (mMRC, CAT), and moderate and severe COPD participants reported worse quality of life (SGRQ), compared with Controls (*p* < 0.05; Table S2).

### 4.4 Correlates of Muscle Oxidative Capacity

Univariate and multivariable linear regression analyses identified correlates of muscle oxidative capacity (*k*). Residual diagnostics improved after log‐transforming *k*, supporting the use of log(*k*). A total of 215 participants were allocated to univariate analysis (Primary Outcome cohort; Figure 1 and Table S3). Of the 87 variables tested in univariate regression, 24 were significantly associated with *k*. The strongest associations were observed for age, DL_CO_ and spirometric variables (Table 2). From these, 11 variables with consistently the highest importance were filtered by machine learning using data from the 162 (75.4%) participants with complete assessments (Analysis cohort; Figure 1).

**TABLE 2:** Univariate regression of association between muscle oxygen consumption recovery rate constant (*k*; proportional to muscle oxidative capacity; log transformed) and clinical and behavioural variables in the Primary Outcome cohort (*n* = 215).

Variable	Units	Estimated coefficient (SE)	*p*
Age	Years	−0.01115 (0.00226)	< 0.0001
DL_CO_	% pred	0.00499 (0.00095)	< 0.0001
FEV_1_	% pred	0.00555 (0.00070)	< 0.0001
FEV_1_	L	0.18641 (0.02346)	< 0.0001
FEV_1_/FVC		0.99424 (0.11276)	< 0.0001
Supplementary O_2_	*N*	−0.42248 (0.06745)	< 0.0001
D‐PPAC amount score	Units	0.00799 (0.00178)	0.00001
VMU/min	Count/min	0.00049 (0.00011)	0.00001
Resting SpO_2_	%	0.03963 (0.00917)	0.00002
SGRQ total score	Units	−0.00387 (0.00091)	0.00003
D‐PPAC total score	Units	0.00798 (0.00195)	0.00006
FVC	L	0.09466 (0.02519)	0.00022
6MWD	m	0.00024 (0.00006)	0.00026
Former smoking	*N*	−0.24483 (0.07111)	0.00069
Any cancer	*N*	−0.22783 (0.06726)	0.00084
Steps/day	Count	0.00002 (0.00001)	0.00094
mMRC	Units	−0.04870 (0.01526)	0.00164
Prostate cancer	*N*	−0.30668 (0.11971)	0.01112
D‐PPAC difficulty score	Units	0.00343 (0.00145)	0.01889
CAT	Units	−0.00609 (0.00250)	0.01547
HADS Anxiety	Units	0.01337 (0.00582)	0.02255
BMI	kg.m^−2^	0.00855 (0.00393)	0.03058
High cholesterol	*N*	−0.10180 (0.04705)	0.03160
Hypertension	*N*	−0.09627 (0.04586)	0.03698
Severe exacerbations last 12 months	*N*	−0.13910 (0.06606)	0.03642
Total number of comorbidities	*N*	−0.02662 (0.01419)	0.06200
Cancer lymphoma	*N*	−0.42151 (0.23787)	0.07784
Mean DBP	mmHg	0.00428 (0.00248)	0.08526
Weight	kg	0.00208 (0.00125)	0.09749
Current smoking; resting HR; race (AA); cancer of the bladder; CHF; osteoarthritis; osteoporosis	—		> 0.10; < 0.20
Gender; ethnicity; height; smoking history; HADS Depression; mean SBP; haemoglobin; recent exercise; AFib; breast cancer; cancer leukaemia; colon cancer; cognitive disorder; CAD; DM; heart attack; hip fracture; kidney disease; liver disease; lung cancer; other type of cancer; PVD; RA; stroke; TIA; uterine cancer	—		> 0.20

Abbreviations: 6MWD, 6‐min walking distance; AA, African American; AFib, atrial fibrillation; BMI, body mass index; CAD, coronary artery disease; CAT, COPD Assessment Test; CHF, congestive heart failure; DBP, diastolic blood pressure; DL_CO_, lung diffusing capacity for carbon monoxide adjusted for [haemoglobin] and altitude; DM, diabetes mellitus; D‐PPAC, Daily‐PROactive Physical Activity instrument in COPD; FEV_1_, forced expiratory volume in 1 s; FVC, forced vital capacity; HADS, Hospital Anxiety and Depression Scale; HR, heart rate; mMRC, modified Medical Research Council Dyspnoea Scale; PVD, peripheral vascular disease; RA, rheumatoid arthritis; Recent exercise, defined as having done exercise in the past 3 weeks (yes/no); SBP, systolic blood pressure; Severe exacerbations, defined as having ≥ 2 severe exacerbations in the past 12 months (yes/no); SGRQ, St. George's Respiratory Questionnaire; SpO_2_, oxygen saturation; TIA, transient ischemic attack; VMU, vector magnitude units.

Table 3 shows results from multivariable regression analysis. FEV_1_%predicted, age and race were significantly associated with *k* (*R* ^2^ = 0.26). Model coefficients equate a 10% lower FEV_1_%predicted to 4.4% lower *k* or 10 years of aging to 9.7% lower *k*. Moreover, individuals of African American race had 11% lower *k* than Non‐Hispanic White peers with the same smoking history. When radiography was included in an exploratory analysis (CT Scan cohort, *n* = 118, 54.9% of study completers; Table S4), FEV_1_%predicted and age remained the only significant correlates of *k* (*R* ^2^ = 0.24; Table 3).

**TABLE 3:** Multivariable regression clinical and behavioural correlates of muscle oxygen consumption recovery rate constant (*k*; proportional to muscle oxidative capacity).

Variable	Units	Analysis cohort (*n* = 162)		CT Scan cohort (*n* = 118)	
		Estimated coefficient	*p*	Estimated coefficient	*p*
FEV_1_	% pred	0.0045	0.0003	0.0047	0.0096
Age	Years	−0.0102	0.0005	−0.0136	0.0003
Race	AA	−0.1059	0.0472	−0.0508	0.4825
Resting SpO_2_	%	0.0153	0.1299	0.0095	0.5692
BMI	kg.m^−2^	0.0049	0.257	0.0023	0.6984
DL_CO_	% pred	−0.001	0.4623	−0.0005	0.7717
Severe exacerbations last 12 months	*N*	0.0294	0.6836	0.0071	0.9398
Current smoking	*N*	−0.0353	0.6732	−0.1301	0.2414
VMU/min	Count/min	< 0.0001	0.7377	< 0.0001	0.5318
Supplemental O_2_	*N*	−0.0327	0.7403	−0.1052	0.4488
PROactive total score	Units	0.0002	0.9302	−0.002	0.5007
Pi10	mm	n/a	n/a	−0.0251	0.7274
% Gas trapping	%	n/a	n/a	0.0024	0.4648

Abbreviations: AA, African American; BMI, body mass index; DL_CO_, lung diffusing capacity for carbon monoxide adjusted for [haemoglobin] and altitude; FEV_1_, forced expiratory volume in 1 s; Pi10, average wall thickness for a hypothetical airway of 10‐mm lumen perimeter on CT; Severe exacerbations, defined as having ≥ 2 severe exacerbations in the past 12 months (yes/no); VMU, vector magnitude units.

When multivariate analysis was stratified by COPD status (Table S5), age remained significantly correlated with *k* in normal spirometry participants (*p* = 0.0255; *n* = 71), while FEV_1_%predicted (*p* = 0.0093) and race (*p* = 0.0051) were correlated with low muscle oxidative capacity in COPD (*n* = 81).

To our surprise, none of our models, including the exploratory analyses, retained any PA‐related variable as a correlate of *k*. To corroborate this finding, we performed a sensitivity analysis on VMU/min (see details in the Supporting Information). First, we removed each potential associated variable in the model (Table 3) and found that VMU/min remained non‐significant (*p* > 0.222–0.984; Table S6). Second, we identified the variables correlated with VMU/min (these were FEV_1_%predicted, D‐PPAC total score, supplementary O_2_ and current smoking) and investigated the significance of VMU/min when two of these correlates were removed at the same time. Interestingly, VMU/min remained non‐significant regardless of which two of the four correlated variables were removed. The lowest *p* value occurred when FEV_1_%predicted and D‐PPAC total score were removed (*p* = 0.100; adjusted *R* ^2^ = 0.18), leaving age as the only significant associated variable (*p* = 0.010; Table S7). Similar results were found when the analysis was run using the CT Scan cohort (*p* = 0.151; adjusted *R* ^2^ = 0.19; Table S8). These analyses confirmed that PA was not a correlate of *k*.

For reference, Table S9 shows that the Per Protocol, Primary Outcome and Analysis cohorts were broadly similar in observed participant characteristics.

## 5 Discussion

This study sought to characterize locomotor muscle oxidative capacity and to identify associated variables in a large group of smokers with and without COPD and never smokers. We hypothesized that low PA and age would be two of the strongest correlates of muscle oxidative impairment. We found that muscle oxidative capacity assessed from the muscle oxygen consumption rate constant (*k*) using NIRS was positively associated with lung function (FEV_1_%predicted), consistent with muscle biopsy studies [5, 6, 24], and negatively associated with age, consistent with some cross‐sectional and longitudinal studies of aging [25, 26, 27]. These results were corroborated in a stratified exploratory analysis based on COPD status, where age was retained as a correlate of *k* in normal spirometry controls, and FEV_1_%predicted and race were correlated with *k* in COPD. However, our regression models demonstrated that free‐living physical activities or COPD radiographic manifestations were not significant correlates of locomotor muscle oxidative capacity.

Muscle oxidative capacity is a key determinant of exercise capacity in health and COPD; it responds to endurance exercise training, contributing to improved exercise tolerance and symptom relief [28, 29]. Impaired mitochondrial oxidative capacity also associates with increased free radical production, greater rates of systemic protein and DNA oxidation [30, 31] and increased circulating diacylglycerides and triacylglycerides in severe COPD [32]. In our Primary Outcome cohort, we found that muscle oxidative capacity was 17.9% lower in mild COPD, and 34%–37% lower in severe COPD, compared with normal spirometry smokers. The magnitude of these impairments was similar to previous reports in smaller cohorts [6, 33].

In absence of smoking or COPD, loss of muscle oxidative capacity is typically ascribed to aging and/or low PA. Some argue that muscle oxidative capacity loss may be protected by PA, such that models accounting for the lower PA associated with older age can explain much of muscle oxidative function loss [34, 35]. It is striking that several studies of older individuals do not find this association [27, 36, 37]. The Study of Muscle Mobility and Aging (SOMMA), which enrolled 697 community‐dwelling elderly (≥ 70 years) with 4‐m gait speed ≥ 0.6 m.s^−1^, showed significant association between accelerometer‐derived PA and muscle oxidative capacity after age and sex adjustment but not after accounting for other covariates including race, BMI and smoking status [27]. Additionally, in SOMMA, increasing age was associated with reduced muscle oxidative capacity only in males and adjusting for PA had negligible impact on this association. Similarly, in our study (both when we included all participants or when stratified by COPD status), muscle oxidative impairment could not be explained by a reduction in PA. Despite identifying VMU/min and D‐PPAC total score as significant correlates in univariate regression, in multivariable regression no activity variable was retained as a *k* correlate. This was further explored in a post hoc sensitivity analysis, which consistently confirmed that PA was not associated with *k* in our study. Our finding of no association between PA and muscle oxidative capacity in COPD is supported by two recent small studies of nine and 12 participants with COPD and activity‐matched controls that found PA could not explain differences in muscle citrate synthase activity, mitochondrial respiratory function or oxidative stress in quadriceps biopsies [38, 39]. In fact, in those studies, as in our 162 participants, spirometric variables were the major correlate of muscle oxidative impairment.

Another feature of our data supporting that low PA may not mediate low muscle oxidative capacity in COPD is that smokers with PRISm had significantly reduced activity behaviours: both VMU/min and steps/day were similar between PRISm and moderate and severe COPD, but muscle oxidative capacity in PRISm was not different from Controls (Figure 2a). Normal muscle oxidative capacity in PRISm, despite reduced PA, also counters an activity‐based explanation for muscle oxidative impairment in COPD. Our results seem to suggest that, in PRISm, the underlying mechanisms responsible for low physical function, increased dyspnoea and morbidity [40] that characterize this transitory clinical state [41] arise from mechanisms independent of mitochondrial impairment.

While cigarette smoke contains many mitotoxic components that may directly impair muscle mitochondrial function (e.g., carbon monoxide and cyanide), we found no association of muscle oxidative impairment with smoke exposure per se in normal spirometry participants (Figure 3a,b) [42, 43, 44]. This may be because exposure (≥ 1 cigarette within 2 h) was too small, that activity of inhibited proteins of the mitochondrial respiratory chain (e.g., cytochrome c oxidase) is normally in excess and therefore reduced enzyme activity did not reduce overall oxidative capacity, or that typical smoke exposure levels do not directly affect skeletal muscle mitochondrial function. Nevertheless, smoke exposure per se could not explain muscle oxidative function impairments observed in this study.

African American race also associated with lower *k* (*p* = 0.0472). To our knowledge, this is the largest study of muscle oxidative capacity in African Americans (*n* = 99), and our results indicate that African Americans with COPD had 11% lower *k* than Non‐Hispanic Whites adjusted for FEV_1_%predicted and age. This difference in *k* could not be explained by low PA: neither steps/day (*p* = 0.169) nor VMU/min (*p* = 0.136) differed between the two race groups. We speculate that race‐specific differences in Type II muscle fibre expression and Hb + Mb concentration between African Americans and Non‐Hispanic Whites might account for the differences observed. Previous studies report that African Americans have a greater proportion of Type II fibres, which may contribute to the observed lower muscle oxidative capacity, lower whole‐body aerobic power and greater risk for cardiometabolic diseases than Non‐Hispanic White peers [45, 46].

FEV_1_%predicted, age and race were identified as correlates of muscle oxidative capacity but only explained ~26% of the variance. Our model showed that FEV_1_%predicted was the strongest correlate of *k* (*p* = 0.0003; Table 3) and that 10% lower FEV_1_%predicted equated to 4.4% lower *k*, i.e., about a fifth of the average decline between normal spirometry and severe COPD at FEV_1_ < 50% predicted. This contradicts previous suggestions that lung function, in itself, is not a major factor responsible for lower limb muscle dysfunction in COPD [9]. While we did not address how pulmonary obstruction might explain locomotor muscle oxidative capacity impairment, we speculate that pulmonary and systemic inflammation in COPD may influence muscle mitochondrial biogenic and angiogenic pathways, for example, PGC‐1α and VEGF expression, and mediate muscle mitochondrial loss, as has been demonstrated in smoke exposed mice [47]. Over several studies assessing COPD muscle using biopsy, low oxidative capacity is a consistent finding, even in two small studies that matched PA between COPD and controls [38, 47]. This suggests that muscle mitochondrial dysfunction may be intrinsic to the disease and not solely related to locomotor muscle disuse [39]. Overall, our findings support the myopathy theory [2, 33] in explaining aetiology of skeletal muscle impairment in COPD.

Although our cohort was among the largest to evaluate correlates of muscle oxidative capacity in COPD, there was 33% missingness in the combination of muscle oxidative capacity and PA data that somewhat reduced statistical power. Future studies should focus on potential mediators of the connection between pulmonary obstruction and muscle oxidative capacity as modifiable variables to protect against muscle function loss. Also, our study design did not include assessment of muscle mass, strength or direct measurement of mitochondrial morphology or enzyme activity. Given the strong association between muscle mass and mortality in COPD [48], identifying whether protecting against muscle oxidative function loss in COPD can protect against muscle atrophy is of considerable interest. The parent study COPDGene only recruited African Americans and Non‐Hispanic Whites (see details in the Supporting Information). While enrolment for our study also included Hispanic (*n* = 3) ethnicity and Asian (*n* = 1) and Other (*n* = 1) race, the sample sizes were too small to retain these categories in statistical analyses. Therefore, our findings will require confirmation in racial and ethnic groups other than African Americans and Non‐Hispanic Whites. Lastly, information about race, ethnicity and medical history were self‐reported, introducing the potential for misclassification.

## 6 Conclusion

Our data indicate that older individuals with stable moderate to severe COPD have an 18%–37% loss in locomotor skeletal muscle oxidative capacity, compared with smokers with normal spirometry. Importantly, this reduction could not be attributed to lower physical activity. Among never smokers and ever smokers with and without COPD, the strongest correlates of lower muscle oxidative capacity were pulmonary obstruction, older age and African American race.

## Funding

This work was supported by National Heart, Lung, and Blood Institute Grant R01HL151452; the Swiss National Science Foundation (SNSF) Grants P300P3_151705 and P300PB_167767; and the Pulmonary Education and Research Foundation. This work was also supported by National Heart, Lung, and Blood Institute Grants U01 HL089897 and U01 HL089856 and by NIH Contract 75N92023D00011. The COPDGene study (NCT00608764) has also been supported by the COPD Foundation through contributions made to an Industry Advisory Committee that has included AstraZeneca, Bayer Pharmaceuticals, Boehringer‐Ingelheim, Genentech, GlaxoSmithKline, Novartis, Pfizer and Sunovion.

## Ethics Statement

Participants were informed about study procedures and risks before giving written informed consent and starting the study protocol. The study was approved by the Institutional Review Board of The Lundquist Institute at Harbor–UCLA Medical Center (20403‐01) and conducted in accordance with the ethical standards laid down in the 1964 Declaration of Helsinki and its later amendments.

## Conflicts of Interest

Alessandra Adami is supported by National Health Institute Grant R01HL151452. Fenghai Duan reports previously receiving consulting fees from EarlyDiagnostics Inc. and Medtronic, outside of the submitted work. Richard Casaburi reports consulting fees from Inogen. Harry Rossiter is supported by grants from the National Health Institute (R01HL151452, R01HL166850, R01HL153460, P50HD098593 and R01DK122767) and the Tobacco Related Disease Research Program (T31IP1666). He reports consulting fees from the National Health Institute RECOVER‐ENERGIZE working group (1OT2HL156812) and is involved in contracted clinical research with United Therapeutics, Genentech, Regeneron, Respira, Mezzion and Intervene Immune. He is a visiting professor at the University of Leeds, UK, and the University of Pavia, Italy. All other authors have nothing to disclose.

## Supporting information


**Table S1:** Comorbidities in the Per Protocol cohort (*n* = 241). Comparison among smokers with normal spirometry (reference group; ‘Controls’), never smokers, preserved ratio impaired spirometry and participants with COPD (subdivided by disease severity). Conditions are listed in alphabetical order.
**Table S2:** Symptoms and quality of life related questionnaires of the Per Protocol cohort (*n* = 241). Comparison among smokers with normal spirometry (reference group; ‘Controls’), never smokers, preserved ratio impaired spirometry and participants with COPD (subdivided by disease severity).
**Table S3:** Participant characteristics of the Analysis cohort (*n* = 162) stratified by clinical status. Comparison is among smokers with normal spirometry (reference group; ‘Controls’), never smokers, preserved ratio impaired spirometry and participants with COPD (subdivided by disease severity stage).
**Table S4:** Pulmonary anatomic variables in the chest CT Scan cohort (*n* = 118). Comparison among smokers with normal spirometry (reference group; ‘Controls’), never smokers, preserved ratio impaired spirometry and participants with COPD (subdivided by disease severity).
**Table S5:** Exploratory multivariable regression of clinical and behavioural correlates of muscle oxygen consumption recovery rate constant (*k*), stratified by COPD status.
**Table S6:** Sensitivity analysis for VMU/min. Each variable included in the multivariate regression model (Table 3) was removed one at a time. VMU/min remains non‐significant in the regression no matter which single variable is excluded (Analysis cohort; *n* = 162).
**Table S7:** Multivariable regression clinical and behavioural correlates of muscle oxygen consumption recovery rate constant (*k*; proportional to muscle oxidative capacity) when FEV_1_%predicted and D‐PPAC were removed after sensitivity analysis reported being correlates of VMU/min (*n* = 162).
**Table S8:** Multivariable regression clinical and behavioural correlates of muscle oxygen consumption recovery rate constant (*k*; proportional to muscle oxidative capacity) on chest CT Scan cohort (*n* = 118). FEV_1_%predicted and D‐PPAC were removed after sensitivity analysis reported being correlates of VMU/min.
**Table S9:** Comparison of participant characteristics among the Per Protocol, Primary Outcome and Analysis cohorts.
**Figure S1:** Residual diagnostics before and after log‐transforming *k*. (a) Model fit on *k*: residuals versus fitted, Q–Q, scale–location and residuals versus leverage/Cook's distance. (b) Model fit on log(*k*) with the same diagnostics. The Q–Q plot shows reduced right‐tail departure and the scale–location plot indicates more constant variance after log transformation, supporting the use of log(*k*).
