# Supplementary material for: Pulmonary Obstruction and Age, Not Activity, Associate With Muscle Oxidative Impairment in Smokers With and Without COPD
Source: J Cachexia Sarcopenia Muscle. 2026 Jan 19;17(1):e70178. doi: 10.1002/jcsm.70178 (PMC12816763; doi:10.1002/jcsm.70178)
Supplement: Supplementary file 1 — Table S1: Comorbidities in the Per Protocol cohort (n = 241). Comparison among smokers with normal spirometry (reference group; ‘Controls’), never smokers, preserved ratio impaired spirometry and participants with COPD (subdivided by disease severity). Conditions are listed in alphabetical order. Table S2: Symptoms and quality of life related questionnaires of the Per Protocol cohort (n = 241). Comparison among smokers with normal spirometry (reference group; ‘Controls’), never smokers, preserved ratio impaired spirometry and participants with COPD (subdivided by disease severity). Table S3: Participant characteristics of the Analysis cohort (n = 162) stratified by clinical status. Comparison is among smokers with normal spirometry (reference group; ‘Controls’), never smokers, preserved ratio impaired spirometry and participants with COPD (subdivided by disease severity stage). Table S4: Pulmonary anatomic variables in the chest CT Scan cohort (n = 118). Comparison among smokers with normal spirometry (reference group; ‘Controls’), never smokers, preserved ratio impaired spirometry and participants with COPD (subdivided by disease severity). Table S5: Exploratory multivariable regression of clinical and behavioural correlates of muscle oxygen consumption recovery rate constant (k), stratified by COPD status. Table S6: Sensitivity analysis for VMU/min. Each variable included in the multivariate regression model (Table 3) was removed one at a time. VMU/min remains non‐significant in the regression no matter which single variable is excluded (Analysis cohort; n = 162). Table S7: Multivariable regression clinical and behavioural correlates of muscle oxygen consumption recovery rate constant (k; proportional to muscle oxidative capacity) when FEV1%predicted and D‐PPAC were removed after sensitivity analysis reported being correlates of VMU/min (n = 162). Table S8: Multivariable regression clinical and behavioural correlates of muscle oxygen consumption recovery rate cons [file JCSM-17-e70178-s001.pdf]

SUPPLEMENTARY FILE

This appendix has been provided by the authors to give readers additional information about their work.

Supplement to: PULMONARY OBSTRUCTION AND AGE, NOT ACTIVITY, ASSOCIATE WITH MUSCLE OXIDATIVE IMPAIRMENT IN SMOKERS WITH AND WITHOUT COPD

Alessandra Adami[1], Fenghai Duan[2], Robert A. Calmelat[3,4], Zeyu Chen[2], Richard Casaburi[3,4], Harry B. Rossiter[3,4]

[1] Department of Kinesiology, College of Health Sciences, University of Rhode Island, Kingston, RI, USA
[2] Department of Biostatistics and Health Data Science, Brown University School of Public Health, Providence, RI, USA
[3] Respiratory Research Center, The Lundquist Institute for Biomedical Innovation at Harbor–UCLA Medical Center, Torrance, CA, USA
[4] Division of Respiratory and Critical Care, Physiology and Medicine, Harbor-UCLA Medical Center, Torrance, CA, USA

Correspondence: Alessandra Adami aadami@uri.edu

METHODS

Participants

The main inclusion criteria for all study participants were: male and female, and aged 45 to 80 years old. Smokers were defined as individuals with ≥10 pack-years history of cigarette smoking. Never smokers were defined as individuals with a lifetime <100 cigarettes, <52 cigars, or <12oz. pipe tobacco smoked; and the absence of a physician diagnosed history of respiratory disease.

For participants enrolled in the COPDGene parent study, being of African American or Non-Hispanic White race was an additional main inclusion criterion, which was not applied to those recruited at the Harbor-UCLA Medical Center Pulmonary outpatient clinic.

The main exclusions for all study participants were: other concomitant respiratory disorder (such as, but not limited to, diffuse bronchiectasis, cystic fibrosis, or interstitial lung disease); lung surgery with removal of a lobe or more (including lung volume reduction and lung transplantation);

lung cancer, known or suspected; bronchoscopic lung volume reduction; pregnancy or suspected pregnancy; uncontrolled cancer, as defined as ongoing radiation therapy, ongoing chemotherapy, narcotics for pain control, or known metastatic disease; history of radiation therapy to the chest (other than radiation for breast cancer); use of antibiotics and/or systemic corticosteroids (new prescription or increased dose) for a COPD exacerbation or any lung infection within the last month; inability to use albuterol; participation in pulmonary rehabilitation within 18 months; inability to perform a spirometry.

For full list of criteria, see protocol available at www.copdgene.org.

Study Design

As part of Phase 2 of the COPDGene[1] (NCT00608764) study protocol, participants completed a series of evaluations including (among others) spirometry, lung diffusing capacity for carbon monoxide (DL_{CO}), inspiratory and expiratory chest CT imaging, six-minute walking distance (6WMD) and questionnaires related to medical and smoking history, occupational and educational background, symptoms (COPD Assessment Test, CAT; modified Medical Research Council Dyspnea scale, mMRC), health-related quality of life (St. George’s Respiratory Questionnaire, SGRQ; 36-Item Short Form Health Survey, SF-36), and anxiety and depression (Hospital Anxiety and Depression Scale, HADS).

The *Muscle Health Study* assessments were the NIRS-based muscle oxidative capacity test, resting pulse oximetry, objectively assessed free-living physical activity (PA) using 7-day triaxial accelerometry (for details on sensor technical characteristics and algorithms, see e.g.[2]), and the Daily-PROactive Physical Activity in COPD (D-PPAC) instrument[3].

Once arriving at the laboratory, participants first completed assessments for the COPDGene study protocol before completing assessments for the *Muscle Health Study*. To avoid potential technical or procedural bias in the results, the 19 participants recruited from Harbor-UCLA Medical Centre Pulmonary outpatient clinic (i.e., not enrolled in COPDGene; Figure 1) were offered the same evaluations and questionnaires, following the same testing order, as those used in the

COPDGene study. All participants were asked to self-report their race and ethnicity using an intake questionnaire.

Pulmonary Function and Imaging

Spirometry was performed in accordance with American Thoracic Society guidelines[4] using a dual-beam Doppler ultrasound-based spirometer (EasyOne Pro, Ndd Medical, Zürich, Switzerland), before and after administration of two puffs of metered dose albuterol sulfate. The FEV_1 and FVC were measured from the greatest values obtained from up to eight maximum expiratory maneuvers, where the greatest two measurements were within 150 mL. Lung diffusing capacity for carbon monoxide (DL_{CO}) was measured in accordance with European Respiratory Society/American Thoracic Society standards for single-breath measurements of carbon monoxide uptake in the lungs[5], and adjusted for haemoglobin and altitude. Relative (%predicted) values were calculated using Global Lung Initiative reference equations[6].

Chest CT scans were performed in the supine position during a single full inspiration (200 mAs) and at end-tidal exhalation (50 mAs) with a 64-detector scanner[1]. Percentage of emphysema (%Emphysema) was defined as percentage of lung voxels with ≤-950 Hounsfield units (HU) attenuation on the inspiratory scan, and %gas trapping as the percentage of the lung volume with voxels ≤-856 HU on the expiratory scan. Airway wall thickening was assessed from the percentage of wall area percent (%Wall area) of 4th and 5th generation airways, and the square root of wall area of a 10-mm perimeter airway (Pi10)[7-9].

Daily-PROactive Physical Activity Instrument in COPD

Participants were asked to reply to the Daily-PROactive Physical Activity instrument in COPD (D-PPAC version)[3], an instrument developed to evaluate the experience of patients with COPD with PA. A paper format of the instrument was used for this study, and it was provided to participant, together with a set of instructions, at the same time the accelerometer was given.

Participants were instructed to record their experiences daily on the same days they wore the accelerometer.

The D-PPAC is a self-administered 7-item questionnaire. Five items relate to the difficulty or limitation in completing daily activities (such as difficulty in getting dressed), and two items relate to the amount of PA done during the day (such as amount of walking). Each item is rated on a 0-4 points scale, and the analysis returns two sub-domain scores, the 'difficulty' and the 'amount' scores, the latter further combined with the daily steps and VMU count data derived from the accelerometer. The final scoring is converted into a Rassh score on a 0 (worst) to 100 (best) scale. The sum of the two sub-domains divided by two provides a total score, ranging from 0 (worse) to 100 (much better)[10]. The D-PPAC instrument is a reliable, valid, and responsive measure in diverse COPD patients[10], with good test-retest reliability in smokers with COPD (ICC ≥0.88)[3].

Comorbidities and Severe Exacerbations

Presence of comorbidities other than COPD were collected using the COPDGene Visit 2 Medical History Form (see copdgene.org; Phase 2 Study Documents). Participants were asked to report whether they had *"ever been told by a physician that they had*" the listed conditions.

The following conditions were considered: atrial fibrillation, breast cancer, cancer leukemia, cancer lymphoma, cancer of the bladder, cancer of the kidney, cancer of the throat and mouth, colon cancer, cognitive disorder, congestive heart failure, connective tissue disorder, coronary artery disease, diabetes mellitus, heart attack, high cholesterol, hip fracture, hypertension, kidney disease, liver disease, lung cancer, osteoarthritis, osteoporosis, other type of cancer, ovarian cancer, pancreatic cancer, peripheral vascular disease, prostate cancer, rheumatoid arthritis, stroke, transient ischemic attack (TIA), uterine cancer. Two additional variables were also calculated: 1) the total number of comorbidities for each participant; 2) the total count of participants within the completers cohort who reported at least one diagnosis of cancer among those considered (named "Any cancer"; see Table S1).

Participants also self-reported whether they had one or more severe exacerbations in the past 12 months. Severe exacerbations were defined as a worsening of respiratory symptoms requiring an emergency room visit or hospitalization. For analysis, we considered the frequency of severe exacerbations, reported as the median[range] (“frequency of severe exacerbations”); and those with 2 or more severe exacerbations, reported as N(%) (“severe exacerbations”).

Statistical Analyses

Data are reported as mean (SD) for continuous variables, counts and percentage for discrete variables. Welch’s ANOVA with Games-Howell *post hoc*, Fisher’s Exact Test of Independence were used to detect differences from smokers with normal spirometry (termed ‘Controls’), as appropriate. No sex-stratified analyses were performed in this study.

To determine variables associated with *k*, after excluding variables with >30% missing data, univariate linear regression analysis was run with log-transformed *k* to satisfy linear regression assumptions. Then, all variables with $p \leq 0.20$ in univariate analysis were further filtered by machine learning-based feature selection methods such that more complex dependencies and interactions among correlates’ effects on the response variable could be taken into consideration. Of the ten comorbidities with $p \leq 0.20$ in univariate analysis, seven (any cancer, cancer of the bladder, high cholesterol, hypertension, osteoarthritis, osteoporosis, total number of comorbidities) were collinear with PA-related variables (either VMU/min and/or steps/day; $p \leq 0.10$) and therefore were excluded from further analysis. The remaining three were excluded for low prevalence within the Per Protocol cohort (cancer lymphoma n=2 (0.8%); congestive heart failure n=15 (6.2%); prostate cancer n=8 (3.3%)).

Three machine learning-based feature selection methods were employed. The first included the Least Absolute Shrinkage and Selection Operator (LASSO), a form of penalized regression incorporating an absolute value penalty term for regression coefficients in its objective function, with the goal to shrink certain coefficients to zero, thus facilitating variable selection within linear models[11]. The other two were tree-based algorithms, Random Forest[12], a method employing

a bagging approach to constructing multiple decision trees in parallel, and the eXtreme Gradient Boosting (XGBoost), a method employing a boosting approach to sequentially building decision trees, each one aiming to correcting errors made by its predecessors[13]. For tree-based algorithms, a series of nodes within a tree are determined to split the feature space into multiple subspaces, and the predicted value for each subspace is calculated. The choice of the splitting variable for a node is typically based on quantitative criteria such as the improvement of node purity or the reduction in mean squared loss. In turn, the variable importance is conveniently measured as a variable's total contribution to the splitting of nodes across all trees according to the quantitative criterion. During the implementation of the three machine learning algorithms, their hyperparameters were selected based on the results of 5-fold cross-validation. The machine learning-based feature selection methods were run on the analysis cohort (n=161; 68.9% of 241 study completers). A *t*-test for unpaired groups showed no differences between the sub-group of 162 participants compared to the 241 who completed the study, for all variables investigated (Table S3 and S9).

By applying both the linear model LASSO and tree-based models Random Forest and XGBoost, we sought to measure the importance of each correlate of *k* in both a linear setting and a tree setting where interactions among correlates were naturally considered. A total of 13 correlates were identified and regarded as the candidate variables to be included in the multivariable linear regression model.

Before fitting the multivariable linear regression model, a variance inflation factor (VIF) for each correlate was computed. According to the screening results, two variables with VIF values larger than 4 were removed from the model to address the collinearity issue. The multicollinearity of these two variables (FEV_1/FVC and number of steps per day) was also confirmed by their extremely high pairwise correlation coefficients with retained correlate (FEV_1%predicted and VMU/min, respectively).

Finally, the remaining top 11 variables identified were entered into a multivariable linear regression to identify correlates of *k*. Due to a relatively large number of participants (43 individuals)

who opted out of CT imaging, a second exploratory multivariable linear regression analysis was performed on participants with complete data including CT imaging (Figure 1). A second exploratory analysis stratified for COPD status was performed including the same set of 11 correlates to identify correlates of *k* in those with and those without COPD (Table S5). For this analysis, the smokers with normal spirometry and never smoker participants were considered as the control group in comparison to those with COPD. Participants with PRISm were excluded from this analysis as, by clinical definition, they have neither COPD nor normal spirometry.

After completing the primary analyses, in order to better understand the influence of significant correlates of *k*, we also explored the association of race (African American or Non-Hispanic white) with steps/day and with VMU/min within the Analysis cohort (n=162). Steps/day and VMU/min were first log-transformed to satisfy linear regression assumptions, and then adjusted for FEV_1%predicted and age, respectively. In addition, we explored the sensitivity of the insignificance of VMU/min in the model. For this, we sequentially excluded each model correlate (Table 3) and investigated its effect on the coefficient and p-value estimation for VMU/min (results reported in Table S6). Next, we evaluated the correlation between VMU/min and each model correlate. For continuous variables, we used Pearson correlation coefficient; for categorical variables, we used 2-sample t-test or ANOVA. This analysis showed that VMU/min was moderately correlated with FEV_1 %predicted, D-PPAC total score, supplemental O_2 use and current smoking. The sensitivity of the insignificance of VMU/min was also evaluated by examining the effect on its p-value estimation when two of the four correlated variables were excluded. By excluding FEV_1 %predicted and D-PPAC total score, VMU/min reached its lowest p-value of 0.100 in the predictive model (R^2=0.18; Table S7), although it remained non-significant. A similar result was found when the CT-scan cohort was used (p=0.151; R^2=0.19; Table S8).

The significance level (α) was set at 0.05. Analyses were performed using SPSS v28 (IBM, Chicago, IL, USA) and R (R 4.0.5); graphical representations with Prism (GraphPad, San Diego, CA, USA).

SUPPLEMENTARY TABLES

Table S1. **Comorbidities in the Per Protocol cohort (n=241)**. Comparison among smokers with normal spirometry (reference group; 'Controls'), never smokers, preserved ratio impaired spirometry, and participants with COPD (subdivided by disease severity). Conditions are listed in alphabetical order.

	PER PROTOCOL COHORT	Never Smokers	Normal Spirometry Smokers (Controls)	PRISm	COPD FEV_1 %predicted			
					> 80	50-79	30-49	< 30
N.	**241**	26	76	21	30	40	35	13
**Condition (N (%))**								
Atrial fibrillation	**7 (2.9)**	0 (0.0)	0 (0.0)	1 (5.0)	0 (0.0)	2 (5.0)	3 (8.6)	1 (7.7)
Any cancer	**28 (11.6)**	1 (3.9)	7 (9.2)	1 (5.0)	3 (10.0)	4 (10.0)	9 (25.7)	3 (23.1)
Breast cancer	**4 (1.7)**	0 (0.0)	1 (1.3)	0 (0.0)	0 (0.0)	1 (2.5)	2 (5.7)	0 (0.0)
Cancer leukemia	**1 (0.4)**	0 (0.0)	0 (0.0)	0 (0.0)	0 (0.0)	1 (2.5)	0 (0.0)	0 (0.0)
Cancer lymphoma	**2 (0.8)**	0 (0.0)	0 (0.0)	0 (0.0)	1 (3.3)	1 (2.5)	0 (0.0)	0 (0.0)
Cancer of the bladder	**2 (0.8)**	0 (0.0)	0 (0.0)	0 (0.0)	1 (3.3)	0 (0.0)	1 (2.9)	0 (0.0)
Cancer of the throat and mouth	**0 (0.0)**	0 (0.0)	0 (0.0)	0 (0.0)	0 (0.0)	0 (0.0)	0 (0.0)	0 (0.0)
Colon cancer	**3 (1.3)**	0 (0.0)	2 (2.6)	0 (0.0)	1 (3.3)	0 (0.0)	0 (0.0)	0 (0.0)
Cognitive disorder	**3 (1.3)**	1 (3.9)	1 (1.3)	0 (0.0)	0 (0.0)	0 (0.0)	1 (2.9)	0 (0.0)
Congestive heart failure	**15 (6.2)**	0 (0.0)	3 (4.0)	3 (15.0)	0 (0.0)	5 (12.5)	3 (8.6)	1 (7.7)
Connective tissue disorder	**0 (0.0)**	0 (0.0)	0 (0.0)	0 (0.0)	0 (0.0)	0 (0.0)	0 (0.0)	0 (0.0)
Coronary artery disease	**11 (4.6)**	1 (3.9)	4 (5.3)	1 (5.0)	1 (3.3)	0 (0.0)	3 (8.6)	1 (7.7)
Diabetes mellitus	**32 (13.3)**	1 (3.9)	12 (15.8)	6 (30.0)	0 (0.0)	5 (12.5)	7 (20.0)	1 (7.7)
Heart attack	**13 (5.4)**	0 (0.0)	5 (6.6)	2 (10.0)	1 (3.3)	2 (5.0)	2 (5.7)	1 (7.7)
High cholesterol	**87 (36.1)**	7 (26.9)	24 (31.6)	6 (30.0)	11 (36.7)	20 (50.0)	14 (40.0)	5 (38.5)
Hip fracture	**8 (3.3)**	0 (0.0)	3 (4.0)	0 (0.0)	1 (3.3)	0 (0.0)	2 (5.7)	2 (15.4)
Hypertension	**121 (50.2)**	8 (30.8)	36 (47.4)	14 (70.0)	11 (36.7)	24 (60.0)	22 (62.9)	6 (46.2)
Kidney cancer	**0 (0.0)**	0 (0.0)	0 (0.0)	0 (0.0)	0 (0.0)	0 (0.0)	0 (0.0)	0 (0.0)
Kidney disease	**4 (1.7)**	1 (3.9)	0 (0.0)	2 (10.0)	0 (0.0)	1 (2.5)	0 (0.0)	0 (0.0)

Liver disease	**9 (3.7)**	0 (0.0)	7 (9.2)	0 (0.0)	1 (3.3)	0 (0.0)	0 (0.0)	1 (7.7)
Lung cancer	**2 (0.8)**	0 (0.0)	0 (0.0)	0 (0.0)	0 (0.0)	0 (0.0)	1 (2.9)	1 (7.7)
Osteoarthritis	**37 (15.4)**	2 (7.7)	12 (15.8)	3 (15.0)	5 (16.7)	6 (15.0)	7 (20.0)	2 (15.4)
Osteoporosis	**21 (8.7)**	1 (3.9)	4 (5.3)	1 (5.0)	2 (6.7)	2 (5.0)	6 (17.1)	5 (38.5)
Other type of cancer	**2 (0.8)**	0 (0.0)	1 (1.3)	0 (0.0)	0 (0.0)	0 (0.0)	1 (2.9)	0 (0.0)
Ovarian cancer	**1 (0.4)**	0 (0.0)	0 (0.0)	1 (5.0)	0 (0.0)	0 (0.0)	0 (0.0)	0 (0.0)
Pancreatic cancer	**0 (0.0)**	0 (0.0)	0 (0.0)	0 (0.0)	0 (0.0)	0 (0.0)	0 (0.0)	0 (0.0)
Peripheral vascular disease	**2 (0.8)**	0 (0.0)	0 (0.0)	0 (0.0)	1 (3.3)	0 (0.0)	0 (0.0)	1 (7.7)
Prostate cancer	**8 (3.3)**	1 (3.9)	2 (2.6)	0 (0.0)	0 (0.0)	1 (2.5)	4 (11.4)	0 (0.0)
Rheumatoid arthritis	**17 (7.1)**	1 (3.9)	5 (6.6)	3 (15.0)	3 (10.0)	3 (7.5)	1 (2.9)	1 (7.7)
Stroke	**3 (1.3)**	0 (0.0)	2 (2.6)	0 (0.0)	0 (0.0)	1 (2.5)	0 (0.0)	0 (0.0)
Transient ischemic attack	**5 (2.1)**	2 (7.7)	1 (1.3)	1 (5.0)	0 (0.0)	1 (2.5)	0 (0.0)	0 (0.0)
Uterine cancer	**3 (1.3)**	0 (0.0)	1 (1.3)	0 (0.0)	0 (0.0)	0 (0.0)	0 (0.0)	2 (15.4)

Data are presented as N (%). FEV_1, forced expiratory volume in 1 second; PRISm, preserved ratio impaired spirometry. Spirometric variables are postbronchodilator.

Table S2. **Symptoms and quality of life related questionnaires of the Per Protocol cohort (n=241)**. Comparison among smokers with normal spirometry (reference group; ‘Controls’), never smokers, preserved ratio impaired spirometry, and participants with COPD (subdivided by disease severity).

	**PER PROTOCOL COHORT**	**Never Smokers**	**Normal Spirometry Smokers (Controls)**	**PRISm**	**COPD FEV_1 %pred**			
					**> 80**	**50-79**	**30-49**	**< 30**
N	**241**	26	76	21	30	40	35	13
mMRC	**1.2 (1.5)**	0.5 (1.0)	0.8 (1.3)	1.3 (1.4)	0.5 (1.0)	1.5 (1.5)	2.8 (1.2) *	3.0 (1.4) *
Effect Size		-0.26		0.52	-0.25	0.75	2.04	2.24
[95% CI]		[-1.04, 0.51]		[-0.57, 1.61]	[-0.99, 0.50]	[-0.12, 1.62]	[1.16, 2.93]	[0.56, 3.91]
CAT	**12.9 (9.0)**	4.5 (6.0) *	12.1 (8.0)	13.1 (10.0)	9.1 (5.8)	15.1 (8.5)	18.8 (8.6) *	20.5 (9.0)
Effect Size		-7.57		0.99	-3.04	3.03	6.69	8.36
[95% CI]		[-12.10, -3.01]		[-6.52, 8.50]	[-7.31, 1.23]	[-2.01, 8.07]	[1.37, 12.00]	[-0.56, 17.30]
SGRQ Total Score	**24.1 (24.2)**	6.5 (14.8)	14.0 (17.1)	22.2 (22.2)	13.8 (18.6)	31.1 (21.9) *	48.0 (20.2) *	58.5 (19.6) *
Effect Size		-7.45		8.25	-0.17	17.10	34.00	44.60
[95% CI]		[-18.20, 3.31]		[-8.41, 24.90]	[-12.20, 11.90]	[4.96, 29.20]	[21.90, 46.00]	[25.10, 64.00]
SGRQ Symptoms score	**24.7 (27.0)**	5.9 (14.7)	14.2 (22.1)	24.0 (29.5)	15.2 (22.6)	37.7 (24.1) *	46.5 (25.5) *	48.8 (21.0) *
Effect Size		-8.30		9.86	0.99	23.50	32.30	34.60
[95% CI]		[-20.00, 3.37]		[-12.20, 31.90]	[-13.80, 15.80]	[9.69, 37.40]	[17.10, 47.60]	[13.50, 55.80]
SGRQ Active score	**35.3 (32.0)**	10.5 (21.8)	23.8 (25.6)	37.0 (29.3)	20.3 (23.5)	43.9 (29.6) *	64.6 (25.1) *	79.3 (19.3) *
Effect Size		-13.30		13.20	-3.46	20.10	40.80	55.50
[95% CI]		[-29.20, 2.66]		[-9.07, 35.50]	[-19.40, 12.40]	[3.32, 36.80]	[25.10, 56.50]	[35.60, 75.40]
SGRQ Impact score	**17.3 (21.8)**	4.4 (11.4)	8.2 (13.3)	13.2 (10.1)	9.6 (17.5)	21.9 (20.3) *	38.7 (21.1) *	49.5 (24.2) *
Effect Size		-3.83		4.96	1.39	13.70	30.50	41.20
[95% CI]		[-12.10, 4.47]		[-9.18, 19.10]	[-9.56, 12.30]	[2.81, 24.50]	[18.50, 42.40]	[17.50, 65.00]
SF-36 PF score	**64.4 (30.6)**	85.4 (22.8)	76.1 (23.5)	58.8 (25.6)	73.0 (23.2)	61.8 (30.9)	39.1 (26.6) *	19.6 (23.7) *
Effect Size		9.33		-17.20	-3.05	-14.30	-36.90	-56.40
[95% CI]		[-6.82, 25.50]		[-36.80, 2.34]	[-18.40, 12.30]	[-31.30, 2.70]	[-52.90, -20.90]	[-80.20, -32.70]
SF-36 RP score	**71.6 (30.7)**	87.7 (22.0)	81.7 (24.5)	65.8 (27.7)	81.7 (27.2)	71.9 (28.8)	48.2 (27.3) *	27.9 (31.1) *
Effect Size		6.08		-15.90	0.01	-9.79	-33.40	-53.80
[95% CI]		[-9.80, 22.00]		[-37.00, 5.21]	[-17.60, 17.60]	[-26.00, 6.46]	[-49.90, -17.00]	[-84.60, -23.00]
SF-36 PCS score	**54.3 (11.2)**	52.2 (8.0)	48.6 (8.9)	42.3 (9.4)	47.7 (9.9)	44.0 (11.8)	35.3 (9.1) *	27.1 (8.8) *
Effect Size		3.62		-6.33	-0.96	-4.59	-13.30	-21.50
[95% CI]		[-2.16, 9.40]		[-13.50, 0.88]	[-7.47, 5.54]	[-11.20, 2.02]	[-20.00, -6.52]	[-32.70, -10.40]
SF-36 MCS score	**50.1 (11.1)**	53.2 (12.0)	49.4 (10.2)	50.4 (14.1)	51.9 (10.5)	50.8 (8.8)	46.9 (9.6)	44.7 (18.9)
HADS Anxiety	**5.2 (3.9)**	4.8 (4.6)	6.0 (3.8)	5.5 (4.5)	4.5 (3.7)	4.1 (3.2)	5.3 (3.7)	5.9 (5.1)

HADS Depression	**4.4 (3.7)**	3.7 (4.3)	4.6 (3.4)	4.1 (3.9)	3.5 (3.5)	3.9 (3.4)	4.9 (3.7)	6.8 (3.5)

Data are presented as mean (SD) if not otherwise specified. PRISm, preserved ratio impaired spirometry; mMRC, modified medical research council dyspnea scale; CAT, COPD assessment test; SGRQ, St. George's respiratory questionnaire; SF-36, Short-form 36-item questionnaire; PF, SF-36 subscale physical functioning; RP, SF-36 subscale role limitations due to physical health; PCS, SF-36 aggregate physical component score; MCS, SF-36 aggregate mental component score; HADS, hospital anxiety and depression scale. Overall tests: Welch's ANOVA. Effect sizes and 95% CIs are shown only when the corresponding overall test is significant. ***** indicates Games–Howell post hoc p<0.05 vs. Controls and a significant overall test.

Table S3. Participant characteristics of the Analysis cohort (n=162) stratified by clinical status. Comparison is among smokers with normal spirometry (reference group; 'Controls'), never smokers, preserved ratio impaired spirometry, and participants with COPD (subdivided by disease severity stage).

	**ANALYSIS COHORT**	**Never Smokers**	**Normal Spirometry Smokers (Controls)**	**PRISm**	**COPD FEV_1 %predicted**			
					**> 80**	**50-79**	**30-49**	**< 30**
N.	162	19	52	10	23	30	17	11
**Personal characteristics**								
Age (yrs)	64.0 (10.0)	57.8 (9.5)	61.1 (9.5)	59.8 (7.1)	70.6 (10.6) *	65.9 (8.3)	69.5 (7.3) *	64.3 (10.3)
Effect Size		-3.30		-1.31	9.54	4.78	8.43	3.22
[95% CI]		[-11.30, 4.73]		[-10.00, 7.40]	[1.53, 17.50]	[-1.32, 10.90]	[1.56, 15.30]	[-8.36, 14.80]
Height (cm)	168.6 (9.9)	166.0 (8.5)	168.3 (9.2)	168.8 (7.0)	168.4 (11.4)	171.3 (10.5)	166.6 (11.5)	169.5 (10.2)
Weight (kg)	79.4 (18.2)	76.8 (16.0)	82.9 (20.5)	89.7 (22.6)	75.0 (13.6)	79.6 (15.9)	73.4 (17.4)	76.0 (19.0)
BMI ($kg.m^{-2}$)	27.9 (5.9)	27.7 (4.7)	28.8 (6.9)	31.2 (6.2)	26.4 (3.3)	27.5 (5.3)	26.5 (5.7)	26.9 (7.7)
Sex (F/M)	87/75	11/8	27/25	6/4	15/8	16/14	10/7	4/7
Race (AA/As/Oth/W)	68/0/0/94	9/0/0/10	34/0/0/18	5/0/0/5	4/0/0/19 *	9/0/0/21 *	4/0/0/13 *	3/0/0/8
Ethnicity (H/Not-H)	1/161	0/19	0/52	0/10	0/23	0/30	0/17	1/10
**Pulmonary function, Medical and smoking history**								
FVC (l)	3.2 (0.9)	3.5 (0.9)	3.4 (0.8)	2.6 (0.4) *	3.7 (0.9)	3.2 (0.9)	2.7 (0.9)	2.2 (0.6) *
Effect Size		0.15		-0.79	0.27	-0.18	-0.73	-1.17
[95% CI]		[-0.60, 0.90]		[-1.33, -0.25]	[-0.42, 0.96]	[-0.80, 0.45]	[-1.50, 0.03]	[-1.88, -0.46]
FEV_1 (l)	2.1 (0.9)	2.9 (0.7)	2.7 (0.6)	1.9 (0.3) *	2.3 (0.6)	1.9 (0.5) *	1.0 (0.3) *	0.6 (0.2) *
Effect Size		0.19		-0.75	-0.35	-0.78	-1.69	-2.08
[95% CI]		[-0.42, 0.79]		[-1.12, -0.37]	[-0.83, 0.12]	[-1.16, -0.40]	[-2.05, -1.34]	[-2.40, -1.77]
FEV_1/FVC	66 (18)	0.8 (0.1)	0.8 (0.1)	0.8 (0.0) *	0.6 (0.1) *	0.6 (0.1) *	0.4 (0.1) *	0.3 (0.1) *
Effect Size		0.02		-0.05	-0.16	-0.20	-0.42	-0.52
[95% CI]		[-0.03, 0.07]		[-0.09, -0.01]	[-0.21, -0.11]	[-0.24, -0.16]	[-0.48, -0.36]	[-0.60, -0.45]
FEV_1 % pred	81.4 (29.3)	107.2 (13.7)	103.0 (13.0)	71.5 (8.3) *	93.9 (12.2)	68.1 (10.2) *	38.5 (5.3) *	21.1 (4.7) *
Effect Size		4.14		-31.60	-9.16	-35.00	-64.60	-82.00
[95% CI]		[-7.27, 15.60]		[-42.00, -21.10]	[-18.80, 0.44]	[-42.80, -27.10]	[-71.30, -57.80]	[-89.00, -74.90]
DL_{CO} % pred	70.6 (24.0)	90.7 (10.3)	80.6 (18.0)	66.0 (15.5)	74.8 (21.3)	67.5 (24.3)	42.5 (13.3) *	36.1 (14.5) *
Effect Size		10.10		-14.70	-5.79	-13.10	-38.10	-44.60
[95% CI]		[-0.44, 20.50]		[-33.50, 4.11]	[-21.60, 10.10]	[-28.80, 2.53]	[-50.80, -25.40]	[-61.30, -27.90]

Hemoglobin (g/dL)	13.7 (1.4)	13.7 (1.2)	13.6 (1.2)	13.6 (1.4)	13.5 (1.1)	13.8 (1.5)	13.6 (2.1)	14.2 (1.9)
Resting SpO_2 (%)	97.4 (2.6)	98.7 (0.5)	98.2 (1.3)	96.6 (4.0)	97.5 (2.5)	97.4 (1.4)	96.2 (2.6)	94.1 (6.0)
Effect Size		0.45		-1.63	-0.71	-0.86	-2.05	-4.14
[95% CI]		[-0.19, 1.09]		[-6.33, 3.07]	[-2.49, 1.07]	[-1.81, 0.08]	[-4.23, 0.13]	[-10.70, 2.39]
Supplementary O_2 (N (%))	18 (11.1)	0 (0)	0 (0)	0 (0)	1 (4)	0 (0)	8 (47) *	9 (82) *
Smoking duration (yrs)	37.0 (9.5)	-	34.7 (9.6)	38.8 (9.1)	38.0 (9.2)	38.9 (8.9)	40.3 (10.4)	34.1 (8.3)
Smoking history (pack-yrs)	44.9 (22.4)	-	39.5 (20.1)	42.3 (20.4)	42.1 (18.5)	53.2 (25.9)	55.1 (19.7)	40.0 (28.4)
Smoking status (NS/CS/FS)	20/89/53	19/0/0 *	0/29/23	0/5/5	0/5/18 *	0/9/21	0/5/12	0/0/11 *
Severe exacerbation (N (%))	20 (12.4)	2 (10.5)	3 (5.8)	0 (0.0)	0 (0.0)	4 (13.3)	7 (41.2) *	4 (36.4)
Frequency severe exacerbation (N median [min-max])	0 [0-6]	0 [0-1]	0 [0-3]	0 [0-2]	0 [0-2]	0 [0-2]	0 [0-2]	0 [0-6]
Effect Size		-0.04		0.20	-0.01	0.30	0.49	1.00
[95% CI]		[-0.31, 0.22]		[-0.59, 1.00]	[-0.35, 0.33]	[-0.16, 0.76]	[-0.18, 1.16]	[-1.11, 3.10]
**Functional performance and Activity**								
BODE	1.7 (2.3)	0.4 (0.7)	0.8 (1.1)	1.6 (1.6)	0.5 (1.1)	1.5 (1.5)	5.3 (1.9) *	7.4 (1.4) *
Effect Size		-0.39		0.79	-0.31	0.69	4.46	6.57
[95% CI]		[-1.07, 0.30]		[-1.07, 2.66]	[-1.16, 0.54]	[-0.29, 1.67]	[2.77, 6.15]	[4.60, 8.53]
6MWD (m)	406 (106)	512 (93) *	402 (85)	419 (69)	448 (99)	408 (61)	342 (108)	227 (97) *
Effect Size		110.36		17.10	46.05	6.89	-59.63	-174.16
[95% CI]		[33.30, 187.50]		[-66.60, 100.80]	[-28.00, 120.00]	[-42.30, 56.00]	[-152.4, 33.1]	[-289.5, -58.9]
Recent exercise (N (%))	97 (59.9)	15 (79.0%)	34 (65.4%)	5 (50.0%)	14 (60.9%)	18 (60.0%)	13 (38.2%)	4 (36.4%)
D-PPAC Total score	63.2 (12.8)	74.9 (0.4) *	65.6 (10.0)	62.1 (8.4)	65.8 (10.6)	64.5 (10.8)	50.4 (10.2) *	43.5 (11.7) *
Effect Size		9.31		-3.51	0.27	-1.07	-15.20	-22.10
[95% CI]		[1.32, 17.30]		[-13.70, 6.71]	[-7.79, 8.33]	[-8.41, 6.26]	[-24.20, -6.17]	[-35.10, -9.10]
D-PPAC Amount score	49.6 (13.6)	57.4 (11.7)	51.9 (12.3)	51.1 (9.6)	54.4 (8.9)	48.9 (12.1)	41.1 (12.8)	29.2 (15.6) *
Effect Size		5.52		-0.75	2.50	-2.91	-10.80	-22.70
[95% CI]		[-4.43, 15.50]		[-12.50, 11.00]	[-5.19, 10.20]	[-11.40, 5.59]	[-22.10, 0.52]	[-40.00, -5.30]
D-PPAC Difficulty score	78.2 (16.3)	92.4 (12.1) *	79.3 (16.3)	73.0 (16.5)	77.3 (16.4)	80.0 (18.1)	59.7 (11.4) *	57.7 (10.0) *
Effect Size		13.10		-6.27	-1.96	0.76	-19.60	-21.50
[95% CI]		[2.02, 24.20]		[-26.00, 13.50]	[-14.60, 10.70]	[-11.50, 13.00]	[-30.70, -8.54]	[-33.70, -9.39]

Data are presented as mean (SD) for continuous variables and as counts (frequencies) for categorical variables if not otherwise specified. BMI, body mass index; F, female; M, Male; AA, African American; As, Asian; Oth, other races; W, White Caucasian; H, Hispanic or Latino; Not-H, Not-Hispanic or Latino; FVC, Forced vital capacity; FEV_1, forced expiratory volume in 1 second ; DL_{CO}, Lung diffusing capacity for carbon monoxide adjusted for [hemoglobin] and altitude; SpO_2, oxygen saturation; NS, never smoker; CS, current smoker; FS, former smoker; Severe exacerbations, defined as having ≥2 severe exacerbations in the past 12 months (Yes/No); BODE, index derived from BMI, airflow obstruction, dyspnea and exercise (6-minute walk distance); 6MWD, 6-minute walking distance (in meters); Recent exercise, defined as having done exercise in the past 3 weeks (Yes/No); D-PPAC, Daily PROActive Physical Activity instrument in COPD (score 0-100). Spirometric variables are postbronchodilator. Overall tests: Welch's ANOVA (continuous) and Fisher's exact (categorical; Monte Carlo p-values). Effect sizes and 95% CIs are shown only when the corresponding overall test is significant. * For continuous variables: indicates Games–Howell post hoc $p < 0.05$ vs. Controls and a significant overall test. For categorical variables: indicates Fisher's exact post hoc $p<0.05/6$ (Bonferroni) vs. Controls and a significant overall test.

Table S4. **Pulmonary anatomic variables in the chest CT scan cohort (n=118)**. Comparison among smokers with normal spirometry (reference group; 'Controls'), never smokers, preserved ratio impaired spirometry, and participants with COPD (subdivided by disease severity).

					**COPD FEV_1 %pred**			
	**CT-SCAN COHORT**	**Never Smokers**	**Normal Spirometry Smokers (Controls)**	**PRISm**	**> 80**	**50-79**	**30-49**	**< 30**
N	**118**	16	68	16	21	33	17	4
%Emphysema	**17.3 (13.4)**	7.1 (7.2)	10.7 (7.2)	8.8 (6.2)	20.9 (11.8)	20.9 (12.6)	38.8 (8.9) *	42.3 (7.2) *
Effect Size		3.63		-0.03	1.80	4.24	14.50	26.30
[95% CI]		[-3.00, 10.30]		[-0.87, 0.81]	[-0.22, 3.82]	[-0.26, 8.74]	[4.85, 24.20]	[4.20, 48.40]
%Gas Trapping	**20.5 (18.8)**	10.8 (9.8)	10.0 (8.6)	8.0 (7.6)	22.5 (12.1) *	25.9 (16.1) *	52.6 (14.4) *	65.5 (7.0) *
Effect Size		1.78		-1.95	12.50	15.90	40.90	55.50
[95% CI]		[-9.22, 12.80]		[-12.60, 8.72]	[1.43, 23.60]	[5.00, 26.80]	[24.60, 57.30]	[36.40, 74.50]
%Wall Area	**48.7 (8.0)**	44.5 (6.2)	45.1 (7.3)	55.3 (8.6)	48.5 (7.1)	52.6 (6.7) *	52.8 (7.2) *	55.1 (6.7) *
Effect Size		0.04		10.60	2.50	7.85	7.05	13.00
[95% CI]		[-6.16, 6.23]		[-1.41, 22.60]	[-3.50, 8.50]	[2.84, 12.90]	[0.35, 13.80]	[3.20, 22.80]
Pi10 (mm)	**2.3 (0.5)**	2.0 (0.3)	2.0 (0.4)	2.8 (0.5) *	2.2 (0.4)	2.6 (0.5) *	2.7 (0.5) *	2.9 (0.5) *
Effect Size		0.07		0.90	0.26	0.72	0.73	1.05
[95% CI]		[-0.27, 0.41]		[0.26, 1.54]	[-0.03, 0.56]	[0.40, 1.03]	[0.24, 1.22]	[0.05, 2.06]

Data are presented as mean (SD). PRISm, preserved ratio impaired spirometry; FEV_1, forced expiratory volume in 1 second; Pi10, average wall thickness for a hypothetical airway of 10-mm lumen perimeter on CT. Overall test: Welch's ANOVA. Effect sizes and 95% CIs are shown only when the corresponding overall test is significant. * indicates Games–Howell post hoc $p < 0.05$ vs. Controls and a significant overall test.

Table S5. Exploratory multivariable regression of clinical and behavioral correlates of muscle oxygen consumption recovery rate constant (*k*), stratified by COPD status.

		COPD (n=81)		Normal Spirometry (n=71)	
**Variable**	**Units**	**Estimated Coefficient**	**P Value**	**Estimated Coefficient**	**P Value**
FEV_1	% pred	0.0042	0.0093	0.002	0.5439
Age	years	-0.005	0.1673	-0.0121	0.0255
Race	AA	-0.1869	0.0051	-0.0291	0.8008
Resting SpO_2	%	0.0167	0.1084	0.0278	0.4408
BMI	kg/m^2	0.0049	0.3392	-0.0026	0.7221
DL_{CO}	% pred	-0.0015	0.2974	0.0018	0.5412
Severe exacerbations last 12-month	N.	0.0086	0.9102	0.105	0.4874
Current smoking	N.	0.3053	0.2173	-0.044	0.7157
VMU/min	count/min	0.0003	0.069	-0.0001	0.5761
Supplemental O_2	N.	-0.0426	0.6527	-	-
PROactive total score	units	-0.0036	0.2233	0.0013	0.7624

FEV_1, forced expiratory volume in 1 second; AA, African American race; BMI, body mass index; DL_{CO}, Lung diffusing capacity for carbon monoxide adjusted for [hemoglobin] and altitude; Severe exacerbations, defined as having ≥2 severe exacerbations in the past 12 months (Yes/No); VMU, vector magnitude units.

Table S6. Sensitivity analysis for VMU/min. Each variable included in the multivariate regression model (Table 3) was removed one at a time. VMU/min remains non-significant in the regression no matter which single variable is excluded (Analysis cohort; n=162).

**Variable Excluded**	**VMU/min**	
	Estimated Coefficient	p-value
FEV_1	0.00011	0.3857
Age	0.00015	0.2220
Race	0.00005	0.7075
Resting SpO_2	0.00005	0.6864
BMI	<0.00001	0.9841
DL_{CO}	0.00005	0.6561
Severe exacerbations last 12-months	0.00006	0.6447
Current smoking	0.00004	0.7629
Supplementary O_2	0.00004	0.7224
D-PPAC total score	0.00009	0.4242

FEV_1, forced expiratory volume in 1 second; AA, African American race; SpO_2, oxygen saturation; BMI, body mass index; DL_{CO}, lung diffusing capacity for carbon monoxide adjusted for [hemoglobin] and altitude; Severe exacerbations, defined as having ≥2 severe exacerbations in the past 12 months (Yes/No); VMU, vector magnitude units; D-PPAC, Daily PROActive Physical Activity instrument in COPD

Table S7. Multivariable regression clinical and behavioral correlates of muscle oxygen consumption recovery rate constant (*k*; proportional to muscle oxidative capacity) when FEV_1 %predicted and D-PPAC were removed after sensitivity analysis reported being correlates of VMU/min (n=162).

Variable	Units	Estimated Coefficient	P Value
Age	years	-0.0071	**0.0098**
Supplementary O_2	N	-0.1581	0.0908
VMU/min	count/min	0.0002	0.0996
Resting SpO_2	%	0.0142	0.1664
Race	AA	-0.0636	0.1967
BMI	kg/m^{-2}	0.0047	0.2609
Current smoking	N	-0.0809	0.2721
DL_{CO}	% pred	0.0009	0.4737
Severe exacerbations last 12-months	N	-0.0234	0.7257

AA, African American race; VMU, vector magnitude units; SpO_2, oxygen saturation; BMI, body mass index; DL_{CO}, lung diffusing capacity for carbon monoxide adjusted for [hemoglobin] and altitude; Severe exacerbations, defined as having ≥2 severe exacerbations in the past 12 months (Yes/No).

Table S8. Multivariable regression clinical and behavioral correlates of muscle oxygen consumption recovery rate constant (*k*; proportional to muscle oxidative capacity) on chest CT-scan cohort (n=118). FEV_1 %predicted and D-PPAC were removed after sensitivity analysis reported being correlates of VMU/min.

Variable	Units	Estimated Coefficient	P Value
Age	years	-0.0094	**0.0044**
Pi10	mm	-0.1072	0.0675
VMU/min	count/min	0.0002	0.1512
Supplementary O_2	N	-0.1611	0.2043
Race	AA	-0.0626	0.3309
DL_{CO}	% pred	-0.0002	0.9208
Current smoking	N	-0.0682	0.4625
Gas trapping	%	-0.0021	0.4666
Resting SpO_2	%	0.0120	0.4722
BMI	kg/m^{-2}	0.0031	0.5731
Severe exacerbations last 12-months	N	-0.0046	0.9555

Pi10, average wall thickness for a hypothetical airway of 10-mm lumen perimeter on CT; VMU, vector magnitude units; AA, African American race; DL_{CO}, lung diffusing capacity for carbon monoxide adjusted for [hemoglobin] and altitude; SpO_2, oxygen saturation; BMI, body mass index; Severe exacerbations, defined as having ≥2 severe exacerbations in the past 12 months (Yes/No).

Table S9. Comparison of participant characteristics among the Per Protocol, Primary Outcome, and Analysis cohorts.

	PER PROTOCOL COHORT	PRIMARY OUTCOME COHORT	ANALYSIS COHORT
N.	241	215	162
**Personal characteristics**			
Age (yrs)	63.9 (9.8)	63.4 (9.8)	64.0 (10.0)
Height (cm)	168.8 (9.8)	169.1 (9.8)	168.6 (9.9)
Weight (kg)	80.8 (18.6)	80.1 (18.4)	79.4 (18.2)
BMI ($kg.m^{-2}$)	28.2 (5.9)	27.9 (5.8)	27.9 (5.9)
Sex (F/M)	128/113	107/108	87/75
Race (AA/As/Oth/W)	110/1/1/129	99/1/1/114	68/0/0/94
Ethnicity (H/Not-H)	3/238	3/212	1/161
**Pulmonary function, Medical and smoking history**			
FVC (l)	3.1 (0.9)	3.2 (0.9)	3.2 (0.9)
FEV_1 (l)	2.1 (0.9)	2.1 (0.9)	2.1 (0.9)
FEV_1/FVC	66 (17)	66 (18)	66 (18)
FEV_1 % pred	78.8 (28.5)	79.7 (29.2)	81.4 (29.3)
COPD GOLD (n) (1/2/3/4)	30/40/35/13	26/39/29/13	23/30/17/11
DL_{CO} % pred	70.4 (22.6)	70.9 (23.2)	70.6 (24.0)
Hemoglobin (g/dL)	13.7 (1.4)	13.7 (1.4)	13.7 (1.4)
Resting SpO_2 (%)	97.5 (2.3)	97.5 (2.4)	97.4 (2.6)
Supplementary O_2 (N (%))	26 (10.8)	24 (11.2)	18 (11.1)
Smoking duration (yrs)	37.5 (10.1)	37.2 (9.8)	37.0 (9.5)
Smoking history (pack-yrs)	44.4 (22.7)	45.1 (22.4)	44.9 (22.4)
Smoking status (NS/CS/FS)	27/82/132	26/115/74	20/89/53
Severe exacerbation (N (%))	31 (13.1)	29 (13.7)	20 (12.4)
Frequency severe exacerbation (N median [min-max])	0 [0-6]	0 [0-6]	0 [0-6]
**Functional performance and Activity**			
BODE	1.7 (2.2)	1.6 (2.2)	1.7 (2.3)
6MWD (m)	396 (107)	402 (106)	406 (106)
Recent exercise (N (%))	135 (56.3)	120 (56.1)	97 (59.9)
D-PPAC Total score	62.3 (12.5)	62.8 (12.6)	63.2 (12.8)
Amount score	48.5 (13.6)	48.9 (13.7)	49.6 (13.6)
Difficulty score	76.1 (17.5)	76.6 (17.4)	78.2 (16.3)

Data are mean (SD) if not otherwise specified. BMI, body mass index; F, female; M, Male; AA, African American; As, Asian; Oth, other races; W, White Caucasian; H, Hispanic or Latino; Not-H, Not-Hispanic or Latino; FVC, Forced vital capacity; FEV_1, forced expiratory volume in 1 second; GOLD, Global initiative for chronic obstructive lung disease; DL_{CO}, Lung diffusing capacity for carbon monoxide adjusted for [hemoglobin] and altitude; SpO_2, oxygen saturation; NS, never smoker; CS, current smoker; FS, former smoker; Severe exacerbations, defined as having ≥2 severe exacerbations in the past 12 months (Yes/No); BODE, index derived from BMI, airflow obstruction, dyspnea and exercise (6-minute walk distance); 6MWD, 6-minute walking distance (in meters); Recent exercise, defined as having done exercise in the past 3 weeks (Yes/No); D-PPAC, Daily PROActive Physical Activity instrument in COPD (score 0-100). Spirometric variables are postbronchodilator

SUPPLEMENTARY FIGURES

Figure S1. Residual diagnostics before and after log-transforming *k*. (**a**) Model fit on *k*: residuals vs fitted, Q–Q, scale–location, and residuals vs leverage/Cook's distance. (**b**) Model fit on log(*k*) with the same diagnostics. The Q–Q plot shows reduced right-tail departure and the scale–location plot indicates more constant variance after log transformation, supporting the use of log(*k*).

a)

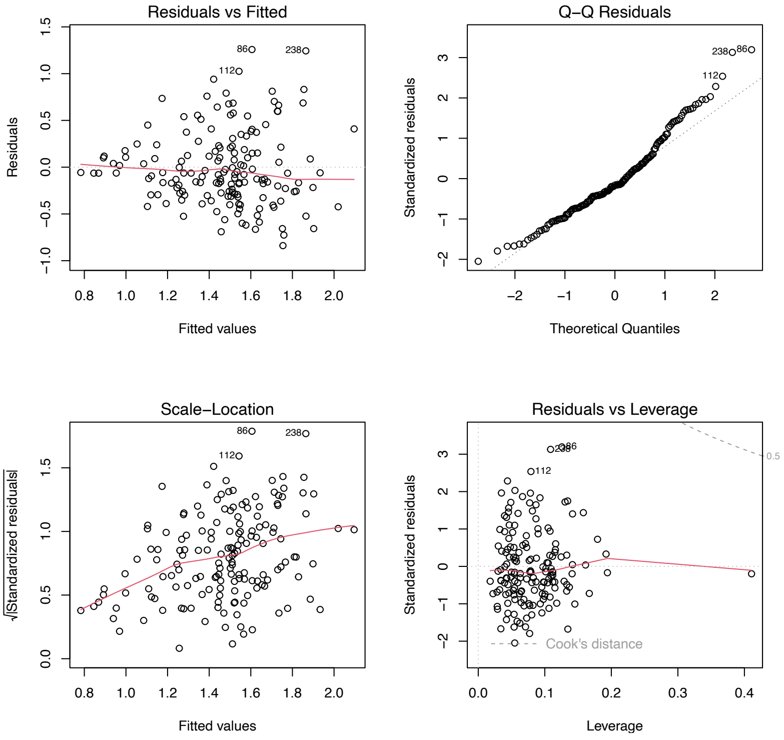

b)

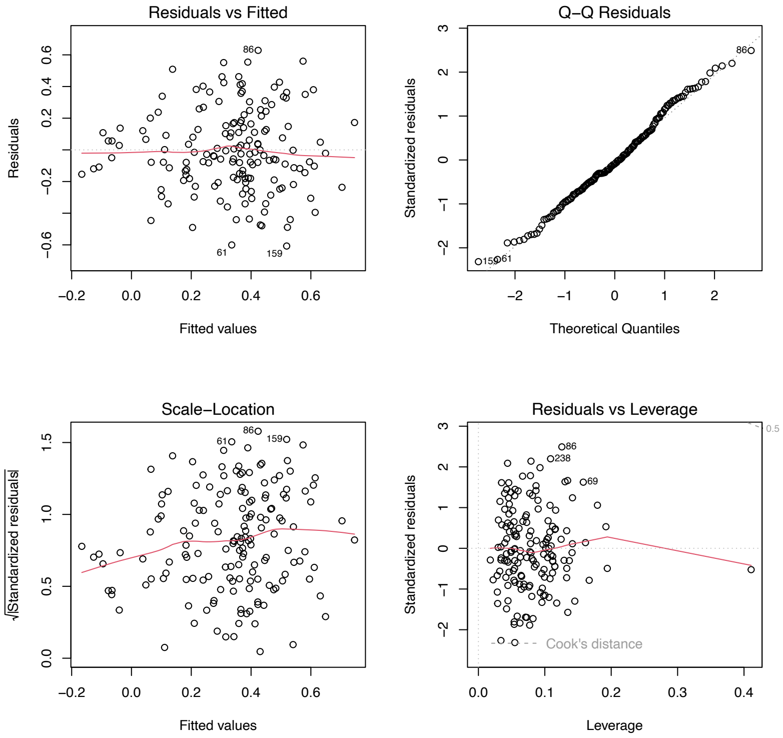

REFERENCES

1. Regan EA, Hokanson JE, Murphy JR, Make B, Lynch DA, Beaty TH, et al. Genetic epidemiology of COPD (COPDGene) study design. COPD. 2010;7:32-43. doi:10.3109/15412550903499522
2. Van Hees VT, Slootmaker SM, De Groot G, Van Mechelen W, Van Lummel RC. Reproducibility of a triaxial seismic accelerometer (DynaPort). Med Sci Sports Exerc. 2009;41:810-7. doi:10.1249/MSS.0b013e31818ff636
3. Gimeno-Santos E, Raste Y, Demeyer H, Louvaris Z, de Jong C, Rabinovich RA, et al. The PROactive instruments to measure physical activity in patients with chronic obstructive pulmonary disease. Eur Respir J. 2015;46:988-1000. doi:10.1183/09031936.00183014
4. Graham BL, Steenbruggen I, Miller MR, Barjaktarevic IZ, Cooper BG, Hall GL, et al. Standardization of Spirometry 2019 Update. An Official American Thoracic Society and European Respiratory Society Technical Statement. Am J Respir Crit Care Med. 2019;200:e70-e88. doi:10.1164/rccm.201908-1590ST
5. Macintyre N, Crapo RO, Viegi G, Johnson DC, van der Grinten CP, Brusasco V, et al. Standardisation of the single-breath determination of carbon monoxide uptake in the lung. Eur Respir J. 2005;26:720-35. doi:10.1183/09031936.05.00034905
6. Miller A, Thornton JC, Warshaw R, Anderson H, Teirstein AS, Selikoff IJ. Single breath diffusing capacity in a representative sample of the population of Michigan, a large industrial state. Predicted values, lower limits of normal, and frequencies of abnormality by smoking history. Am Rev Respir Dis. 1983;127:270-7. doi:10.1164/arrd.1983.127.3.270
7. Nakano Y, Wong JC, de Jong PA, Buzatu L, Nagao T, Coxson HO, et al. The prediction of small airway dimensions using computed tomography. Am J Respir Crit Care Med. 2005;171:142-6. doi:10.1164/rccm.200407-874OC
8. Patel BD, Coxson HO, Pillai SG, Agusti AG, Calverley PM, Donner CF, et al. Airway wall thickening and emphysema show independent familial aggregation in chronic obstructive

pulmonary disease. Am J Respir Crit Care Med. 2008;178:500-5. doi:10.1164/rccm.200801-059OC

9. Martinez CH, Chen YH, Westgate PM, Liu LX, Murray S, Curtis JL, et al. Relationship between quantitative CT metrics and health status and BODE in chronic obstructive pulmonary disease. Thorax. 2012;67:399-406. doi:10.1136/thoraxjnl-2011-201185

10. Garcia-Aymerich J, Puhan MA, Corriol-Rohou S, de Jong C, Demeyer H, Dobbels F, et al. Validity and responsiveness of the Daily- and Clinical visit-PROactive Physical Activity in COPD (D-PPAC and C-PPAC) instruments. Thorax. 2021;76:228-38. doi:10.1136/thoraxjnl-2020-214554

11. Tibshirani R. Regression shrinkage and selection via the Lasso. Journal of the Royal Statistical Society: Series B (Methodological). 1996;58:267-88.

12. Breiman L. Random forests. Machine Learning. 2001;45:5-32.

13. Chen TG, C. XGBoost: A scalable tree boosting system. In KDD ’16: Proceedings of the 22nd ACM SIGKDD International Conference on Knowledge Discovery and Data Mining. 2016;785-94. doi:http://dx.doi.org/10.1145/2939672.2939785
